# A zero power harmonic transponder sensor for ubiquitous wireless μL liquid-volume monitoring

**DOI:** 10.1038/srep18795

**Published:** 2016-01-06

**Authors:** Haiyu Huang, Pai-Yen Chen, Cheng-Hsien Hung, Ranjit Gharpurey, Deji Akinwande

**Affiliations:** 1 Department of Electrical and Computer Engineering, The University of Texas at Austin, Austin, TX 78712, United States of America; 2 Maxim Integrated, Dallas, TX 75240, United States of America; 3 Department of Electrical and Computer Engineering, Wayne State University, Detroit, MI 48202, United States of America

## Abstract

Autonomous liquid-volume monitoring is crucial in ubiquitous healthcare. However, conventional approach is based on either human visual observation or expensive detectors, which are costly for future pervasive monitoring. Here we introduce a novel approach based on passive harmonic transponder antenna sensor and frequency hopping spread spectrum (FHSS) pattern analysis, to provide a very low cost wireless μL-resolution liquid-volume monitoring without battery or digital circuits. In our conceptual demonstration, the harmonic transponder comprises of a passive nonlinear frequency multiplier connected to a metamaterial-inspired 3-D antenna designed to be highly sensitive to the liquid-volume within a confined region. The transponder first receives some FHSS signal from an interrogator, then converts such signal to its harmonic band and re-radiates through the antenna sensor. The harmonic signal is picked up by a sniffer receiver and decoded through pattern analysis of the high dimensional FHSS signal strength data. A robust, zero power, absolute accuracy wireless liquid-volume monitoring is realized in the presence of strong direct coupling, background scatters, distance variance as well as near-field human-body interference. The concepts of passive harmonic transponder sensor, metamaterial-inspired antenna sensor, and FHSS pattern analysis based sensor decoding may help establishing cost-effective, energy-efficient and intelligent wireless pervasive healthcare monitoring platforms.

Like the shift of telephone booth centered telecommunication to ubiquitous wireless communication from last few decades, healthcare is starting a paradigm shift from hospital centered care to ubiquitous care^1^,^2^. Emerging technologies are enabling new terms such as telemedicine, e-hospital, e-health and u-health^1^,^2^,^3^,^4^,^5^. One of the key features of ubiquitous healthcare is wireless pervasive monitoring^1^,^6^. As global demographic trend of aging will result in increasing healthcare workforce shortage^7^,^8^, electronic healthcare monitoring is gradually taking over the role of human-based observation on both diagnostic and therapeutic sides^9^,^10^,^11^,^12^,^13^,^14^. It is predicted that wirelessly networked sensors with not only high sensitivity, but also small size, low power and minimum cost (in fabrication, deployment and maintenance) will receive growing popularity in healthcare industry^13^,^15^.

Liquid is essential of all forms of life; almost all healthcare practices are related to liquid manipulation^16^,^17^. Dynamically and accurately monitoring liquid-volume anywhere anytime will be an important feature in ubiquitous healthcare. For instance, for nutritional or therapeutic liquid intake, the liquid-volume inside a container is usually checked each time for pharmaceutical compliance. For intravenous therapy, it requires the process being monitored to avoid any hazardous incident, such as the air embolism when the medicine is fully dispensed in the drip chamber or syringe. For controlled drug delivery or smart pills, the amount of drug remained in the capsule is a critical feedback information to assure the delivered dosage within certain therapeutic ranges that maximize the efficacy and also minimize side effects^18^,^19^,^20^,^21^. However, conventional monitoring, either for large containers or small syringes, is based on human visual observation. High resolution liquid-volume monitoring for miniaturized containers, on the other hand, are mostly relying on expensive detectors, such as optical sensors^22^. Therefore, low cost automatic real-time wireless monitoring of liquid-volume would be crucial to alleviate the load of healthcare workers and to improve the overall healthcare efficiency.

In wireless sensor networks, battery is a major hurdle to further downsize the sensor node and minimize the cost; thus for short distance applications, passive sensors are preferred over active sensors^23^. Passive radio frequency identification (RFID) transponder sensors have gained attention from academia and industry^24^,^25^,^26^,^27^,^28^,^29^,^30^. Especially, instead of having extra sensor modules, antennas on commercial ultra-high-frequency (UHF) RFID transponders can be used as the sensing agent for beverage liquid-volume monitoring^29^,^30^. However, these RFID transponder antenna sensors suffer from inability of absolute value sensing^29^,^30^. For instance, the background interference (which commonly exists in the indoor environment), the sensing distance, and the input power will affect the sensor output. To achieve high-resolution and reliable wireless liquid-volume sensing, better platforms are desired.

Major challenge also exists for healthcare wireless liquid monitoring in its antenna/sensor design. Unlike beverage bottle monitoring^29^,^30^, commercial UHF RFID transponder antenna would not be suitable for most healthcare liquid monitoring applications, which have much higher miniaturization and sensing resolution requirements. The sensing antenna on the transponder must be specifically designed to serve the applications in healthcare. Metamaterials, which are artificial materials with engineered electromagnetic responses, have been proposed to make various miniaturized antennas based on the substrate^31^, or coverage^32^ of single/double negative materials. Moreover, the metamaterial-inspired electrically small antennas (ESAs), comprising of the LC resonant structures inspired from the unit element of metamaterials, can be more readily designed and physically realized compared to those metamaterial-based ESAs. It has been demonstrated that a driven electrically-small electric or magnetic antenna covered by metamaterial-inspired structures in its near field can be matched to a 50 Ohm source and have high overall efficiency. A similar concept of metamaterial cover or the so-called “cloaking device” has also currently proposed to maximize the received power of a small dipole antenna^33^. In this case, a reactive metamaterial helical cover can control the scattering cross-section of the ESA system associated with the antenna resistance (which inherently has a small value for an ESA) and cancel the reactive energy in the near field of ESA. Therefore, the conjugate matching can be achieved for the ESA system, providing the maximum power efficiency and potentially maximum sensitivity to liquid materials filled inside the antenna.

Here we propose a new sensing mechanism utilizing harmonic transponder sensor with metamaterial-inspired compact three-dimensional (3-D) liquid-sensitive antenna, and frequency hopping spread spectrum (FHSS) pattern analysis to realize passive high resolution absolute accuracy wireless liquid-volume monitoring. Historically, harmonic radar with electronic nonlinear tags has been used for tracking moving insects^34^. In our sensing system, the harmonic transponder sensor converts the received UHF RFID signal at 902–928 MHz band from an interrogator to its second harmonic at 1.804–1.856 GHz band. The harmonic tones containing liquid-volume sensing information, can be potentially detected and decoded by a portable sniffer device (e.g. smart phone with 3G and LTE antenna covering 1.8 GHz) and therefore avoid direct coupling, background scatters and clutters at the fundamental frequencies. In addition, the FHSS feature required by the UHF RFID communication protocol^35^, is utilized to provide high dimensional data for sensing purpose. High dimensional FHSS data enables algorithm based pattern analysis. Just like computer vision^36^,^37^, FHSS pattern analysis provides robust absolute accuracy sensing and decision which is impossible for one dimensional data. The redundant information from extra dimensions can tolerate variation of other factors, such as transponder sensor location and interference from near-field objects. We apply those concepts to realize a wireless medical saline liquid volume monitoring with μL resolution on a metamaterial-inspired ESA, which is, in some sense, similar to a miniaturized monopole antenna with a metamaterial-surface cover^32^,^33^. The high quality-factor (Q-factor) feature of such subwavelength antenna would leak to a strong field localization^31^,^38^ and therefore the ability of reliably and sensibly detecting the liquid-volume within the micro-liter range. This battery and digital circuit free wireless sensing mechanism shows great potential in a broad range of healthcare applications, including pharmaceutical management, drug delivery control, drug compliance assurance, clinical research, as well as other non-healthcare fields which have the need of dynamically monitoring the liquid-volume.

## Results

### Passive harmonic transponder based sensing with FHSS pattern analysis

**Figure 1:**
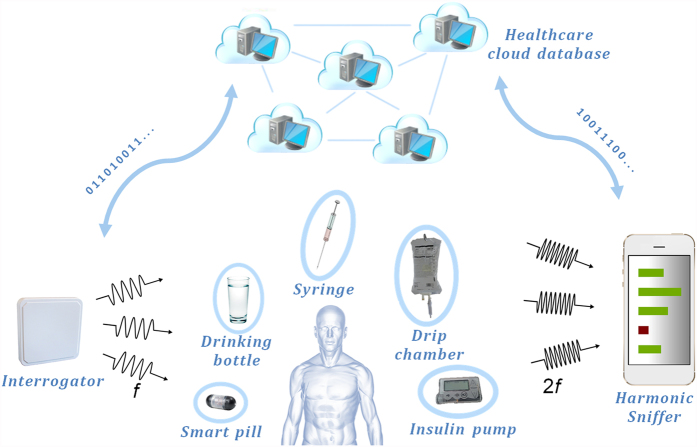
Application of passive harmonic transponder sensor and sniffer based liquid-volume sensing system in a frequency hopped personal area network. In the sensing system, an interrogator transmits frequency hopped signals. Each healthcare liquid container is enclosed with a passive harmonic transponder sensor that converts the signals to their harmonic band and retransmits them through a liquid sensitive 3-D antenna. A portable device may serve as a harmonic sniffer receiver to detect and decode the re-transmitted sensing signals at the harmonic bands from each transponder sensor. Cloud based computation and database is connected to both the interrogator and the harmonic sniffer, serving as the hub for storing the sensing information and managing healthcare liquid manipulations in a whole healthcare facility. Graphic arts of human body and clouds were created by Jo Wozniak with Texas Advanced Computing Center; The Syringe (https://pixabay.com/en/injection-syringe-needle-hypodermic-29845/), smart pill (http://www.madboxpc.com/ipill-la-super-pildora/), drip chamber (https://en.wikipedia.org/wiki/Intravenous_therapy#/media/File:Infuuszakjes.jpg), water glass (http://savagehow.wikispaces.com/How+to+watercolor+a+sunset+or+sunrise) and insulin pump (https://commons.wikimedia.org/wiki/File:Insulin_pump.jpg) are licensed under the Attribution-ShareAlike 3.0 Unported license. The license terms can be found on the following link: https://creativecommons.org/licenses/by-sa/3.0/.

A high level illustration of the proposed passive harmonic transponder sensor and FHSS pattern analysis based liquid-volume sensing system is shown in Fig. 1. An interrogator transmits a frequency hopped signal. Various kinds of liquid containers with different geometries and applications, like bottle, dripping chamber, syringe, drug capsule with controllable drug delivery (smart pill) or insulin pump, can be equipped with passive harmonic transponder sensors to receive signals from the interrogator. The harmonic transponder sensor has a nonlinear module that converts the signal to its harmonic band. Such harmonic output is emitted from a specifically designed 3-D antenna operating at the corresponding harmonic band, and having a radiation property across the band highly sensitive to the liquid-volume in the container. The harmonic signal is then detectable and decodable by a sniffer receiver through analyzing the high dimensional FHSS pattern. In practice, the interrogator could be a 902.5–928.5 MHz UHF RFID reader with 50 frequency hopping channels and a smart phone or tablet with receiving antenna already covering 1.8–1.9 GHz could be utilized as the sniffer receiver. As most UHF RFID interrogators and smart phones are connected to internet and accessible to cloud based computation and database, the passive harmonic transponder sensors can be indirectly linked to some sensing cloud and support complicated healthcare management infrastructures.

**Figure 2:**
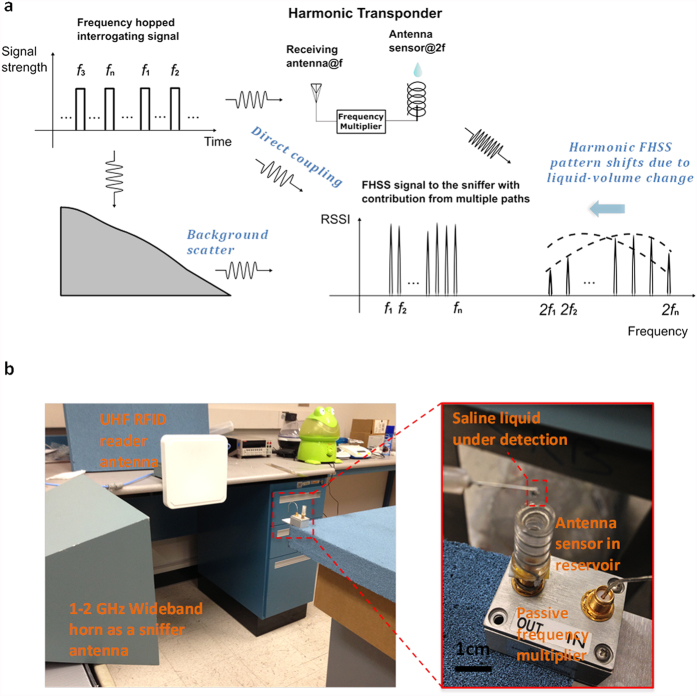
Passive harmonic transponder sensor based liquid-volume sensing system. (**a**) Sensing mechanism: The signal from the interrogator is frequency hopping among a set of channels [f_1_, f_2_ … f_n_] with constant signal strength; at the sniffer receiver end, the FHSS pattern of the RSSI array profile over the harmonic channels represents the overall antenna gain of the sensing antenna, which is affected by the liquid-volume shift inside the container. (**b**) Experimental setup for conceptual demonstration: picture (left) of the passive wireless liquid monitoring system tested in rich scattering indoor environment; and picture (right) of a fabricated enhance wave meta-material cloak antenna sensor, which can detect μL level liquid applied from a syringe (with 30-gauge needle) to the enclosed PDMS packaged cylinder container.

The detailed sensing mechanism is shown in Fig. 2a. The interrogating signal has a pseudo-random hopping sequence covering channels [f_1_, f_2_ … f_n_] with constant signal strength. Assuming other than the sensitive harmonic antenna, the rest of the components in the system, such as interrogator antenna, transponder sensor fundamental antenna, frequency multiplier, and sniffer receiver antenna, all have relatively invariant frequency responses, then the FHSS pattern over channels [2f_1_, 2f_2_ … 2f_n_] of the received signal strength indicator (RSSI) at the sniffer will discretely represent the sensing antenna resonant profile from 2f_1_ to 2f_n_, which is sensitive to the liquid-volume shift. Frequency conversion on the transponder sensor is necessary because at the fundamental frequencies there is strong direct coupling from the interrogator to the sniffer receiver, as well as background scattering in the environment. On the other hand, at the second harmonic band the signals are exclusively from the harmonic transponder, thus are much cleaner with high signal-to-noise ratio. Also, comparing to sensors with one-dimensional data which relies on the signal amplitude, the high dimensional RSSI vector provides abundant information to enable pattern analysis based decoding, therefore achieves robust and absolute accuracy liquid-volume sensing.

### Metamaterial-inspired antenna sensor

**Figure 3:**
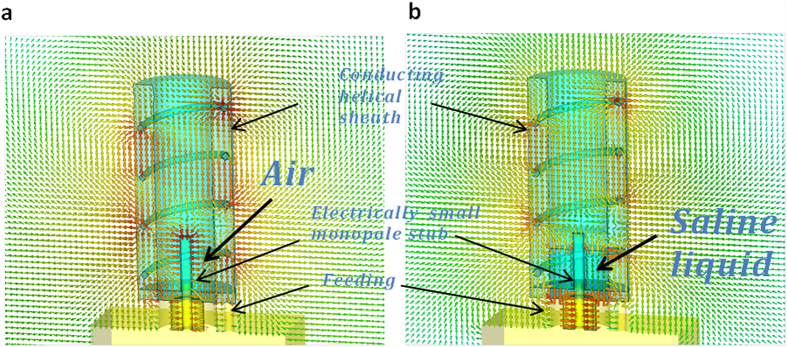
Metamaterial inspired antenna sensor. (**a**) Simulated electric field vectors for an empty metamaterial-inspired antenna sensor. (**b**) With saline liquid filled around the electrically small monopole stub, the field distribution changes because the resonant frequency is highly sensitive to the liquid-volume.

The liquid sensitivity boosting and sensor miniaturization are achieved by a metamaterial-inspired ESA design, where a short-stub monopole is surrounded by a helical coverage^31^ as a near-field resonant parasitic^39^. A conducting ground plane leads to the half-sized monopole and the helical coverage, due to the mirror effect. The advantage of this structure is shown through an electric field distribution comparison of a monopole with (shown in Fig. S1a) and without such helical coverage (shown in Fig. S1b). In fact, for an asymmetric helical structure with direction of helix reversed at the center^40^, the magnetic dipole component is canceled at the resonance, and only the electric dipolar radiation polarized parallel to the helix axis is presented, which is somehow similar to the lowest order spherical harmonic (TM_0_ mode) of a polarized subwavelength epsilon-negative (ENG) or plasmonic rod from the Mie scattering theory^31^, creating the inductance necessary to establish the resonance within the electrically small structure. The small monopole stub at the center of the helical structure therefore provides a strong coupling between the coaxial feeding and resonant mode of the reactive cover. From the full-wave simulations in Fig. 3a, we found the electric fields are anti-phase, intense and uniform within the helix, showing striking similarities to the resonant mode of an ENG-metamaterial rod^40^ or a plasmonic particle at optical frequency^41^. Here the spatially distributed inductance of helical coverage may tune out the large capacitance of the electrically-small monopole stub when immersed in the liquid solution as shown in Fig. 3b. Since ESAs are typically characterized by large values of Q-factor and the dielectric liquid drug inside the coverage is immersed in strongly localized electric fields, according to the cavity perturbation theory^21^, its resonant frequency may sensibly depends on the liquid-volume. Especially, the local liquid-volume sensitivity around the rod is boosted. For our antenna sensor designed at 1.85 GHz, 1 μL liquid-volume change may cause a 2.5 MHz resonant frequency shift, according to the return loss simulation result in Fig. S1d. In addition, like any ESA, the spatial radiation pattern of the antenna sensor is a typical isotropic one as shown in Fig. S1c. The behavior of the meta-material inspired antenna sensor can be also understood through a RLC resonant circuit model as shown in the supplementary material and Fig. S2. The measured S11 (as shown in Fig. S3a) of the antenna sensor under different liquid volumes validates the sensor behavior predicted by the simulation and model calculation.

The sensor realized here is a generic prototype in order to show a design approach toward a high resolution liquid-volume sensor around a miniaturized reservoir. For specific applications, design needs to base on the geometry and application environment. For example, for implanted applications in a fluid and tissue surrounding environment, the dielectric constant of the surrounding materials needs to be take into consideration^21^. In addition, the conductivity of the liquid material affects the quality factor of the sensor, but is independent of its resonance. From the measured data in Fig. S3b we can see that the sensor can tolerate a large range of liquid conductivity variation, and is suitable for most practical medical liquid from non-saline to very highly saline. Also the same sensor may be used to identify a liquid material if fixing the liquid volume, which is beyond the application scope of this work.

### Experimental realization of passive μL liquid-volume monitoring

**Figure 4:**
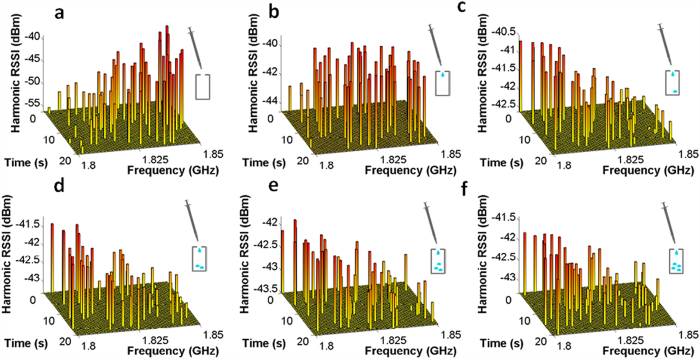
Demonstration of passive harmonic transponder sensor wireless liquid monitoring with frequency hopped 902.75–927.75 MHz UHF RFID signal. (**a**) When there is no liquid in the reservoir, the 3-D bar chart shows the RSSI array on both time and frequency domain. (**b**–**f**) The 3-D bar chart of the second harmonic RSSI array when there is 10,20,30,40,50 μL PBS solution in the reservoir, respectively.

**Figure 5:**
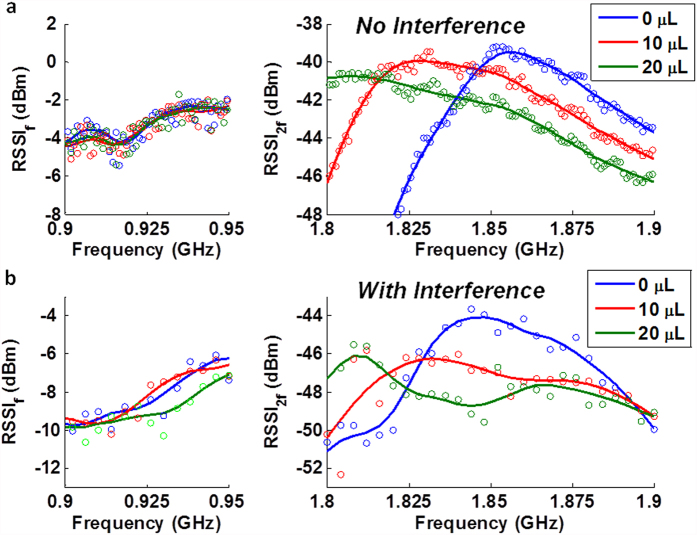
The measured harmonic RSSI array at both fundamental and harmonic bands. (**a**) With no human body interference; Interrogating signal is ASK with 0.5 MHz hopping channel width. **(b)** With moving human body interference from only a few centimeters away; Interrogating signal is unmodulated carrier with 2 MHz hopping channel width.

Figure 2b shows the harmonic transponder sensor prototype with fabricated metamaterial-inspired sensing antenna and the experimental setup in a rich scattering indoor environment (Detailed signal characterization of the system and harmonic generation is shown in Figs S4–6). The measured sensing data is summarized in Fig. 4., in turns of the 3-D bar charts of harmonic RSSI against both time and frequency under frequency hopping. The frequency hopping sequence is set to be randomly permutated among the 50 channels from 902.75–927.75 MHz and the resolution bandwidth of the sniffer receiver is set to be 0.5 MHz to match the ASK signal channel width^42^. The received harmonic signal thus has the same hopping pattern at harmonic frequencies from 1.8055–1.8555 GHz. Variations of the FHSS patterns can be observed from Fig. 4a–c. The FHSS pattern has a peak around 1.855 GHz when the liquid-volume is 0 μL (Fig. 4a). By adding 10 μL and 20 μL of liquid, the second harmonic peak shifts to 1.83 GHz (Fig. 4b) and 1.805 GHz (Fig. 4e), respectively. Therefore, if the channel index of the harmonic peak is used as a digital output, the sensor resolution is 20 μL/50 = 0.4 μL. However, the pattern stays almost constant when the liquid-volume reaches 30, 40 and 50 μL, as shown in in Fig. 4d–f, indicating a sensing range up to 20 μL. Figure 5a compares the measured RSSI array by the sniffer receiver over the fundamental band and the harmonic band. The RSSI array variation at the fundamental band is hardly perceptible because the received power at those frequencies is dominated by the direct coupling from the RFID reader antenna and the scattering from nearby objects. In contrast, as the liquid-volume increased from 0 μL to 20 μL, the RSSI array at the second harmonic shows distinct profile variations. This validates the advantage of the harmonic transponder over conventional transponders as a passive sensing platform.

### High dimensional data based passive wireless sensing with absolute accuracy

The high dimensional received harmonic RSSI array sensing data contains redundancy for liquid-volume sensing decision. In fact only after a few hops a correct decision of the peak frequency can be made through the regression algorithm (see supplementary video demo). Such “free” redundancy is provided by the FHSS feature which is a requirement of the UHF RFID protocol. Similar to computer vision which turns the imaging sensing data into insight, here more redundancy means better robustness liquid-volume sensing with absolute accuracy that tolerates variations.

In body area networks, the human body is a time varying interferer that may affect the wireless communication and severely degrade the amplitude-modulation based sensor performance. This is an important issue improved by the high-dimensional RSSI array. As compared in Fig. 5a,b, when a constantly moving human body is in close proximity (a few centimeters away) to the passive sensor, the harmonic signal strength degrades and the RSSI deviation gets worse, but the redundant information of the multi-dimensional output ensures the overall profile of the RSSI peak channel index is preserved for each liquid volume level. In addition, we have verified is its independence of interrogating signal source. The UHF RFID reader may send either ASK signal or unmodulated carrier signal, and the actual channel gap may vary as the hopping pattern may skip some channels. For Fig. 5a the interrogating signal is ASK with hopping 0.5 MHz channel width, while for Fig. 5b the interrogating signal is unmodulated carrier with hopping channel width of 2 MHz. Comparison of other modulation types including FSK,PSK,AM and FM is included insee supplementary materials. Such tolerance of signal variation may lead to cross-system or cross-protocol interoperability (Bluetooth, Wifi etc.) for the ubiquitous wireless healthcare monitoring.

**Figure 6:**
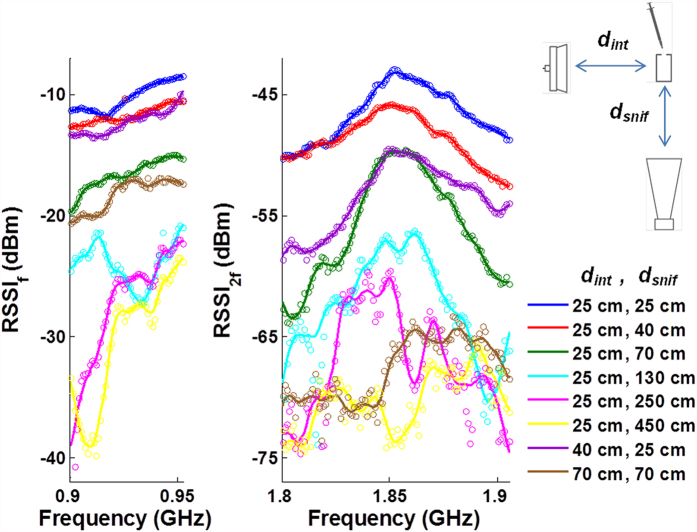
RSSI array variation for different sniffer-to-transponder distance as well as interrogator-to-transponder distance, with liquid-volume fixed at 0 μL. The peak frequency is consistent around 1.85 GHz, indicating distance independence.

Another critical problem being resolved is distance dependence, which was considered as a major drawback of passive RFID tag sensors^29^. As shown in Fig. 6, when the sniffer receiver is moved away from the sensor, even though the absolute signal strength decreases drastically, the FHSS pattern and the peak frequency of harmonic RSSI array are largely preserved. Communication channel fading becomes observable at a 2.5 m sniffer-to-sensor distance. As the sensor output is a high dimensional output, the regression algorithm is still able retrieve the correct liquid-volume decision. At 4.5 m the channel fading is severe enough to bring the signal-to-noise-ratio down to a level that liquid-volume sensing is no longer valid. Figure 6 also shows variation of interrogator-to-transponder distance. In our demonstration the interrogator generates a 10 dBm power signal. According to Friis’ equation^43^, if with maximum permitted UHF RFID interrogating signal power at 30 dBm, the sensing distance range can potentially cover most indoor applications. However, in practice, boosting the power to 30 dBm could associate with power amplifier unit that may result in worse second harmonic distortion for the interrogating signal. Therefore, it is important to ensure that the sensor signal from the transponder still dominates the harmonic band.

### Performance improvement through post-processing algorithm

**Figure 7:**
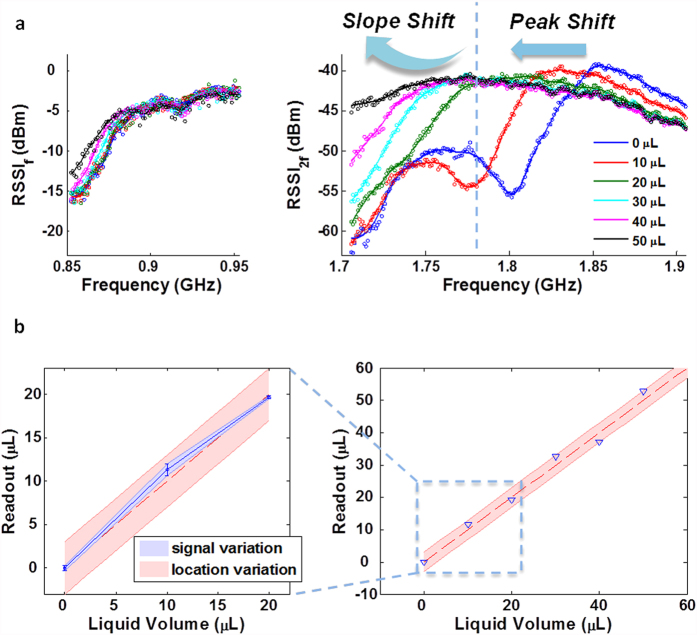
Comprehensive demonstration of the sensing system with wider frequency hopping range covering the whole global UHF RFID band. (**a**) RSSI array variation at both fundamental tone (left) and second harmonic tone (right) for liquid-volume applied from 0 μL to 50 μL. The Harmonic FHSS pattern variation can be divided into a peak shift region (0 to 30 μL), and a slope shift region (30 to 50 μL). (**b**) Summary of the sensor performance after the algorithm based pattern analysis on the high dimensional data. The subplot on the left specifies the accuracies due to signal variation and location variation, which location variation induced error dominates.

The 20 μL sensor measurement range can be improved by increasing the hopping bandwidth and applying more complex pattern analysis algorithm. The 902–927 MHz band is defined by EPC C1-Gen2 protocol in United States; while worldwide operating band ranges from 840 MHz to 960 MHz and some commercial RFID readers cover the whole UHF RFID band. For that a more comprehensive measurement result is shown in Fig. 7a, with the FHSS covering 850 MHz to 950 MHz without being restricted by specific local protocol. For liquid volume of 30 μL to 50 μL, FHSS pattern variation is observable in the wider spectrum from 1.7 to 1.8 GHz. However, such pattern variation is tilt slope shift instead of peak shift. This phenomenon is caused by the narrow band effect from the interrogator and transponder fundamental tone antenna in the signal path, which is verified through the signal characterization along the path (by comparing Figs S4 and S5). Substituting those antennas with wider band antennas can potentially eliminate such effect and increase sensor dynamic range. Here, instead of using high cost wideband antennas, we take the advantage of its multi-dimensional nature of the output and utilize a robust local regression and narrow band effect compensation algorithm (see method) for the FHSS pattern analysis and get an overall linear sensing performance with an extended sensing range up to 50 μL. The final sensor readout is shown in Fig. 7b. Figure 7b also summarized the sensor accuracy estimated through statistical measurement results. For fixed location scenario, interrogating signal variation contributes to the sensor error, and the measured accuracy is 0.6 uL (3% if consider 0 to 20 uL as the sensing range). Consider location variation (for sniffer-transponder distance less than 3 meters), the accuracy is 2.4 uL (12% for 20 uL sensing range), which dominates over the signal variation. Strong interference can make the accuracy even worse, but the data is not included here as human body interference is not able to quantify. In conclusion, with 0.4 uL resolution, 50 uL and approximately 10% accuracy, the proposed sensing system is suitable for very low cost pervasive liquid-level monitoring with moderate sensing accuracy.

## Discussion

We have successfully realized zero-power wireless liquid-volume monitoring at the μL-scale. The proposed sensor system employs a new concept of FHSS harmonic transponder antenna sensor, which uses frequency-hopped signal to retrieve the response spectrum of a harmonic transponder antenna. The harmonic transponder sensor receives frequency-hoped signal and coverts it to its second order harmonic, avoiding strong direct coupling and background scatters in the environment. The transponder sensing antenna has a metamaterial-inspired, 3-D electrically-small resonant parasitic structure, for which the frequency response is designed to be highly sensitive to the liquid-volume inside. The sensing system is experimentally demonstrated through a UHF RFID protocol compatible prototype. In addition, the absolute-accuracy sensing capability that tolerates distance variation as well as human body proximity interference is an advantageous feature very important in on-body and implanted applications.

Our demonstration presents a digital circuit free prototype at UHF RFID band, but the sensing feature can be integrated with existing technologies and applications. For example, having a conventional RFID IC chip on the transponder sensor, identification may be performed at the same time with harmonic sensing, where the reflected ID data path is still picked up by the interrogator. Alternatively, if using a radar-like highly directional interrogator, localization information of the transponder sensor may also be retrieved liquid-volume. In this case, communication and synchronization between the interrogator and the sniffer is necessary for comprehensive data fusion of liquid-volume, ID and location information of the passive sensor networks.

In addition, UHF RFID is not the only suitable platform, other communication protocols with spread spectrum techniques, such as Wifi, Bluetooth, and LTE, may all have their own versions of transponder sensors and sniffers. Last but not least, techniques from computer vision, such as supervised learning algorithm can be also applied to the high dimensional sensor data as a more advanced post-processing for the sensing decision. Overall, this work will have great potential to promote wirelessly networked ubiquitous monitoring in healthcare and other industrial fields.

## Methods

### Antenna sensor simulation

Practical design of the antenna sensor with metamaterial-inspired (or “cloak”) structure is based on frequency-domain finite-element method (FEM) simulation using the commercial software CST microwave studio software^44^. In the full-wave simulation, the package material with relative permittivity of 2.8 and loss tangent of 0.0035, were considered, and the short monopole and helical coverage are made of coppers with conductivity of 5.8 × 10^7^ S/m. The liquid material in the simulation has a permittivity of 80, corresponding to commonly used biomedical liquids such as phosphate buffered saline (PBS) buffer. The sensor nominal operation frequency is determined by the selection of helix parameters such as helical radius, number of turns and helical pitch angle determines the operating frequency range, and the length of short stub also plays an important role to fine tune the antenna resistance and match the frequency multiplier circuit^32^. The short stub length and its relative position to the helical cover also affect the local sensitivity and resolution of the sensor. In practice, the short stub length can be empirically fine-tuned for optimized matching and performance.

### Antenna sensor fabrication

The metamaterial-inspired (or “cloak”) structure is assembled within Polydimethylsiloxane (PDMS)^21^ package to also form a liquid reservoir. The metal wire antenna is fabricated using selective laser sintering based mold process in order to precisely produce the helical structure^45^. Starting with a reservoir mold and a package mold, the PDMS mixture liquids are filled in with the metal helical structure. PDMS with the molds are first cured at room temperature in a desiccator for more than one hour and then in a 125 °C oven for about 40 minutes^21^. Putting the whole assembly in an environment with temperature below 0 °C will help release the molds. The passive frequency multiplier selected here is a MiniCircuit MK-5^46^ with a low conversion loss from 20 MHz to 2 GHz. This frequency multiplier was packaged with metal shield which can also serve as the antenna ground plane. One end of the metal wire has to be connected to the ground, either through soldering it to the metal cover of frequency multiplier or through Ohmic contact of the metal wire with the outside thread of the SMA connector on the frequency multiplier (The second way makes the sensor reservoir easily replaceable and disposable).

### Experimental setup of the sensing system demo

The fabricated antenna was connected to the output port of the passive frequency multiplier. The input port of the frequency multiplier connected to a folded monopole antenna at fundamental tone was characterized together with the frequency multiplier as shown in Fig. S3. The transmitting module in the demonstration is a commercial RFID interrogator antenna connected to a signal generator, which emulates the frequency hopped ASK signal source based on UHF RFID protocol. A wideband horn antenna connected to a PXA spectrum analyzer to detect and characterize the harmonic sensing signal reradiated from the transponder. Here the spectrum analyzer is emulating the function of the sniffer receiver circuit for conceptual demonstration. If using a portable device, such circuit can be designed and integrated with other frontend circuits in the same frequency band such as LTE receiver, and share the antenna and possibly the analog frontend. The sniffer antenna is chosen to be wideband in order to compare the signals at both fundamental and harmonic bands. Standard medical purpose PBS solution (electrical conductivity measured as 22 mS/cm) was injected to the PDMS reservoir through a 30-gauge syringe needle with 10 μL per liquid load as the smallest increment during the liquid-volume sensor demonstration. The measurement was done in a typical indoor environment with wireless connections such as Wifi, LTE and GSM. Two smartphones and one laptop device were turned on in the vicinity of the test setup.

### Generation of interrogating signals

A link layer code programmed in Labview is controlling the signal generator to produce a narrow band ASK signal with its carrier frequency hopping at least every 400 ms, compatible with the Class 1, generation 2 (C1Gen2) UHF RFID protocol^42^. The allocated band in U.S. is 902.5–928.5 MHz with 500 kHz channel width (excluding the two guarding bands) with totally 50 channels for frequency hopping^42^. The hopping is following a predefined random permutations pattern. The data on the interrogating signal for test is a pseudo random binary sequence with 100 kHz symbol rate. As a result, the output harmonic signal will be hopping among 1.805–1.857 GHz with 1 MHz channel width following the same permutation order. The hopping range can be changed through the Labview program, for example, from 850 MHz to 950 MHz for wide band demonstration. In addition, the modulation type can be also set to FSK, PSK, FM, AM or PM for more general demonstration.

### Algorithm based FHSS pattern analysis

The high dimensional RSSI array data can be first applied with some regression model to generate a regression curve, for example, here a robust local regression with weighted linear least squares and a 2^nd^ degree polynomial model^47^. This regression assigns zero weight to data outside six mean absolute deviations to rule out abrupt interference from the environment. The peak channel frequency f_peak_ of the regression curve is then identified through a peak detection algorithm. Subsequently, a compensated peak channel frequency f_peak_comp_ is calculated through a narrow-band-effect compensation algorithm:


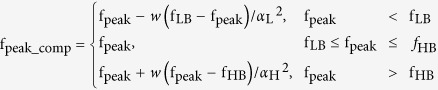


where f_LB_ and f_HB_ defines the narrow band effect boundary for the peak shift and slope shift regions, *w* is a constant weight coefficient, 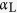 and 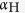
are the first order polynomial fitting coefficients for the data in the range of (f_peak_ –Δf, f_peak_) and (f_peak_, f_peak_ + Δf), correspondingly, where Δf can be assigned with a reasonable value, e.g. 25 MHz, for slope tangent calculation. Having the compensated peak channel frequency, the final step is to map f_peak_comp_ to the corresponding resonant frequency based on the calibrated liquid-volume dependence curve associated with the particular antenna sensor used, and get the readout liquid-volume value in μL.

## Additional Information

**How to cite this article**: Huang, H. *et al.* A zero power harmonic transponder sensor for ubiquitous wireless µL liquid-volume monitoring. *Sci. Rep.* **6**, 18795; doi: 10.1038/srep18795 (2016).

## Supplementary Material

Supplementary Information

Supplementary Video S1
