# Supplementary material for: A zero power harmonic transponder sensor for ubiquitous wireless μL liquid-volume monitoring
Source: Sci Rep. 2016 Jan 6;6:18795. doi: 10.1038/srep18795 (PMC4702130; doi:10.1038/srep18795)
Supplement: Supplementary Information [file srep18795-s1.doc]

**A zero power harmonic transponder sensor for ubiquitous wireless μL liquid-volume monitoring**

Haiyu Huang^1,2^, Pai-Yen Chen^3^, Cheng-Hsien Hung^1^, Ranjit Gharpurey^1^, Deji Akinwande^1^

## Affiliations

### **Department of Electrical and Computer Engineering, The University of Texas at Austin, Austin, TX 78712 United States of America**

### **Maxim Integrated, Dallas, TX 75240, United States of America**

### **Department of Electrical and Computer Engineering, Wayne State University, Detroit, MI 48202, United States of America**

## Corresponding author

Haiyu Huang, Harryhuang@utexas.edu.

**Equivalent circuit model of meta-material inspired antenna liquid-volume sensor**

The resonant frequency shift behavior of the meta-material inspired near-filed parasitic (in this case the helical cover) antenna can be analyzed through a RLC resonant circuit model^1^. As shown in Fig. S2a, the monopole stub along with its near-filed surrounding structure forms a RLC resonator, in which the resistance *R* and capacitance *C* are affected by the liquid level Δ*V* and liquid permittivity *ε_r_* ^2^. Here Δ*V* is denoted the relative liquid volume inside the sensor reservoir to simplify the expression (Δ*V*=1 when liquid level is full). Both *R* and *C* are divided into two serially connected parts, one has permittivity of 1 as for the empty region, the other has permittivity *ε_r_* as for the liquid occupied region. Because the antenna sensor is designed to be perfectly match to 50 Ω at 1.85 GHz, we could first determine that *R*_0_=50 Ω and 1/(2π
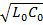
)=1.85 GHz. The value of *C*_0_ and *L*_0_ can be further selected based on the realized quality factor Q of the antenna sensor. Having the initial model parameters, S*_11_* variation of the circuit model can be calculated as shown in Fig. S2b. The complete antenna sensor structure including the effect of liquid level not only on monopole stub but also on the helical cover^3^ can be modeled as a more complicated circuit as shown in Fig. S2c, in which the simple RLC resonator represents the monopole stub, while the helical cover is modeled as an impedance transformer with its own RLC circuit (*R*_1_,*L*_1_,*C*_1_) feeding to a load Z_L_ representing the overall load looking from the helical cover which radiates to the free space. Here the mutual inductance *M* is modeling the near-field evanescent wave coupling between the monopole stub and the helical cover. The calculated S*_21_* under different liquid level are shown in Fig. S2d.

**Independence of passive sensing on interrogating signal source variation**

Transmitting signal power, signal modulation type, and signal frequency hopping channel width variation will not significantly affect the sensing result and performance. The measurement data in Fig. 5a is associated with ASK-modulated signal, while that in Fig. 5b is single tone sinusoidal signal. It is evidently seen that they present similar profiles and peak frequencies on the RSSI array. A more detailed comparison of the RSSI array with popular modulation types (single tone, ASK, FSK, PSK, AM and FM) is shown in Fig. S7. The sensing data indicates that the peak channel is at 1.85 GHz regardless of modulation type. In Fig. 5b, the frequency hopping channel width is 2 MHz comparing to 0.5 MHz in Fig. 5a, and the resolution bandwidth of the receiver is 3 MHz comparing to 100 kHz in Fig. 5b, the degradation on the sensor performance due to channel width variation remains unnoticeable.

**a**

**b**


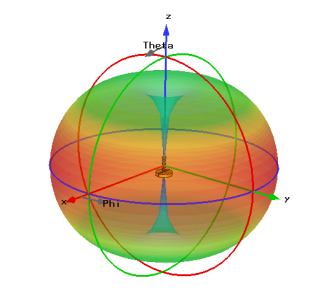


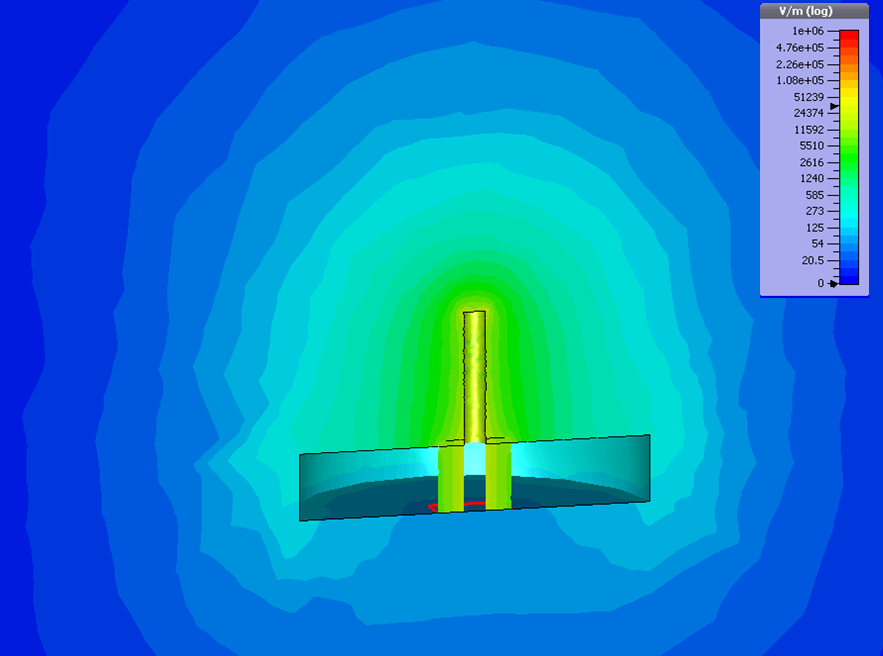

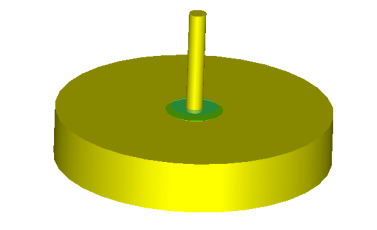

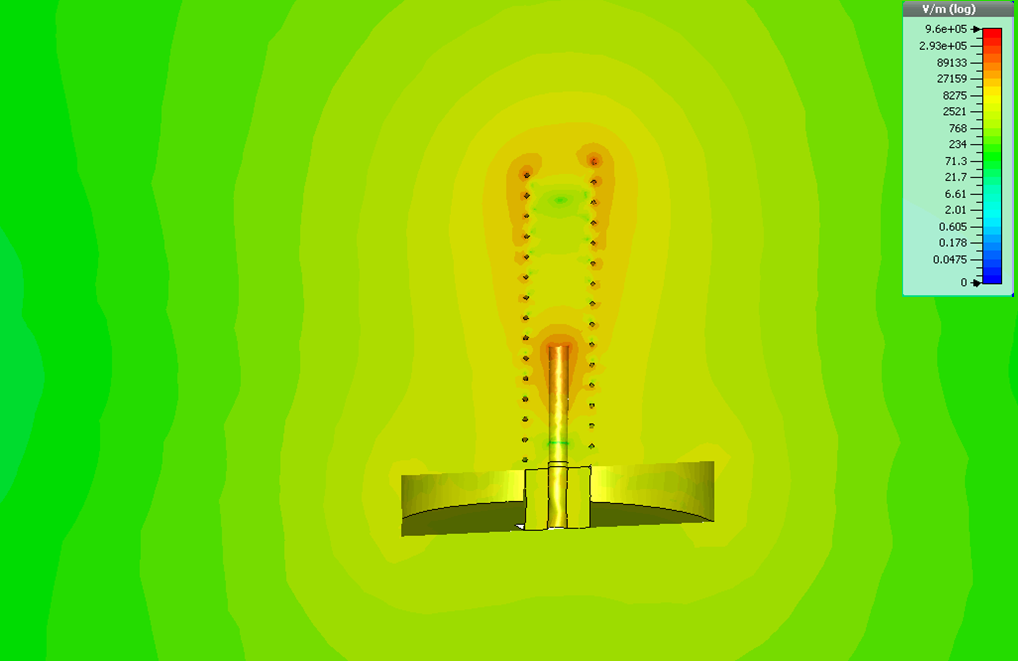

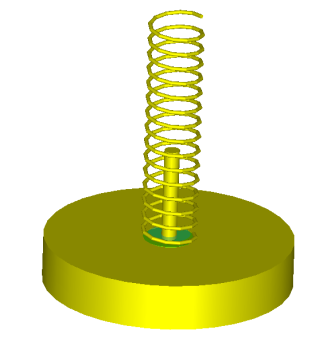


**c**

**d**

***Covered***

***Uncovered***

**Figure S1. Simulation of metamaterial inspired antenna sensor.** **(a)** Geometry and simulated field distribution of monopole stud without the metamaterial-surface coverage. **(b)** is similar to (a), but for a cloaked monopole stud; now the field is significantly enhanced and strongly confined within the region around the stud, boosting sensitivity on detecting the amount of liquid filled inside the metamaterial-surface coverage. **(c)** The 3-D view of far field pattern. **(d)** The return loss variation of the antenna sensor under different liquid levels.


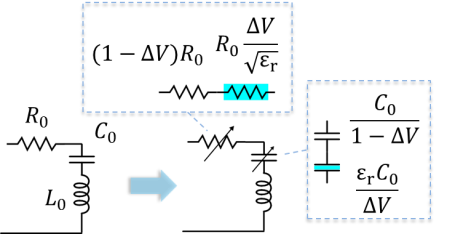


**a**

**c**

**d**

**b**


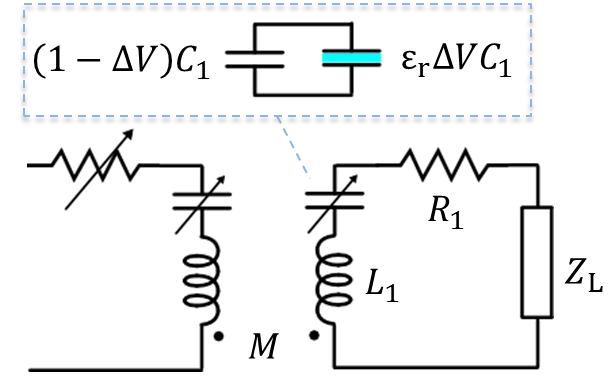


**Figure S2: Equivalent resonant circuit model of the antenna sensor.** (a) The monopole stub along with its surrounding structure forms a RLC resonator, in which *R* and *C* are affected by the liquid level Δ*V* and liquid permittivity *ε_r_*. (b) Calculated S*_11_* of the circuit model with varying liquid level. (c) A complete circuit model including both monopole stub and helical cover structure. (d) The calculated S*_21_* (port 2 as Z_L_) of the circuit in (c) affected by liquid level change.

**a**

**b**

**Figure S3: Measured S11 of an antenna sensor**: (a) filled with different volumes of PBS solution with an electrical conductivity of 22 mS/cm. (b) filled with fixed volume (about 35 μL) of liquid with different liquid conductivities; the increasing PBS conductivity decreases the quality factor of the antenna sensor. (Due to the syringe control accuracy during the test, the actual volume for the water data is slightly different from the PBS data, causing the slight difference in their resonant frequency).


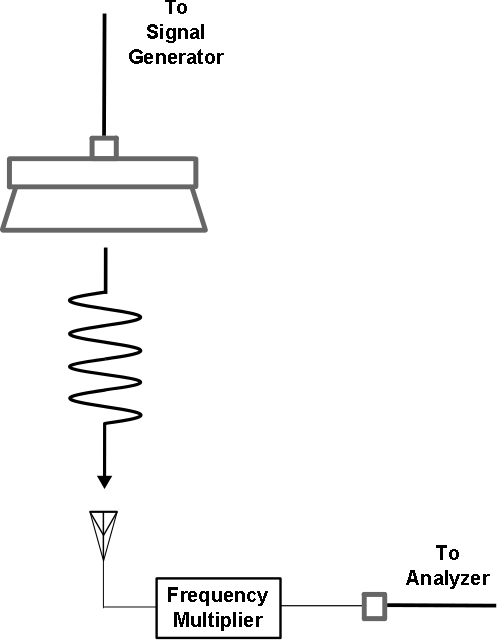


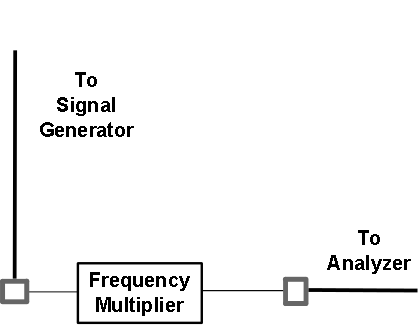


**b**

**a**


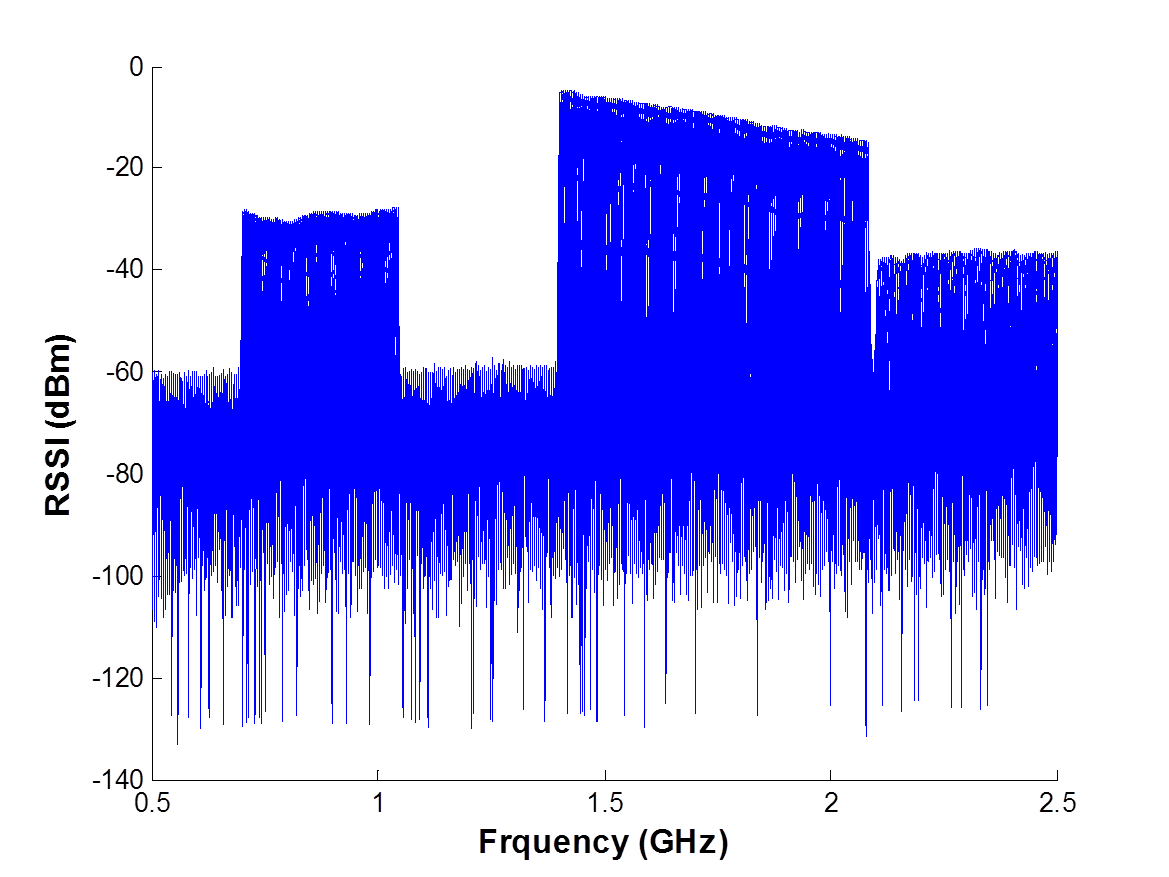
**
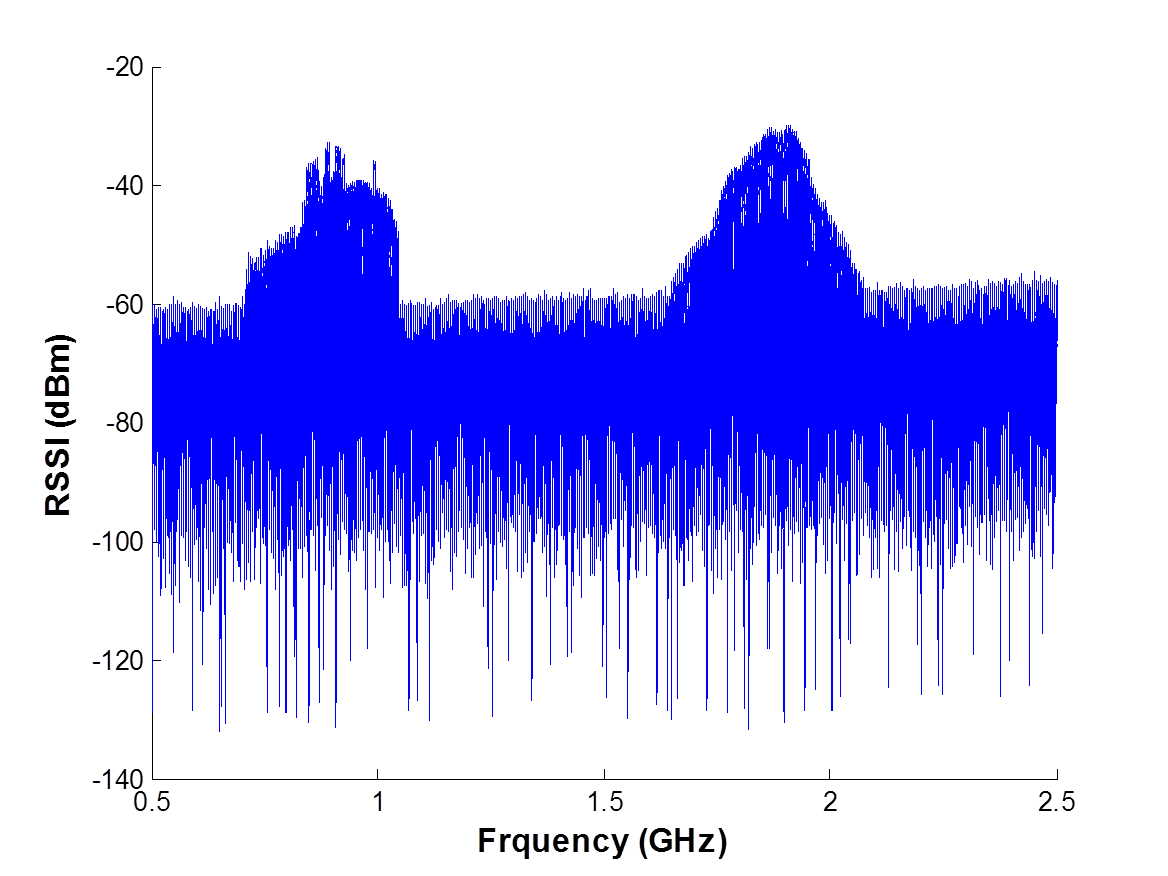
**

**Figure S4.** **Signal characterization with interrogator and tag fundamental antenna.** **(a)** Test of the frequency multiplier under frequency sweep from 700 MHz – 1.05 GHz, as a reference; the overall conversion gain (including cable loss and other system loss) over the second harmonic band is from -18 dB to -27 dB (10 dBm from signal generator); the gain at fundamental bands and third harmonic band is around -40 dB and -50 dB, respectively; **(b)** Adding the UHF RFID reader antenna and fundamental tone receiving antenna at the transponder, from the overall spectrum response under frequency sweep, the overall conversion gain generally shows narrow band feature with relatively flat peak around 1.8-1.9 GHz. A coarse 3 MHz resolution bandwidth is used here to allow a faster frequency sweep, so the noise floor levels up to -60 dBm; but it in general will not affect the signal characterization.


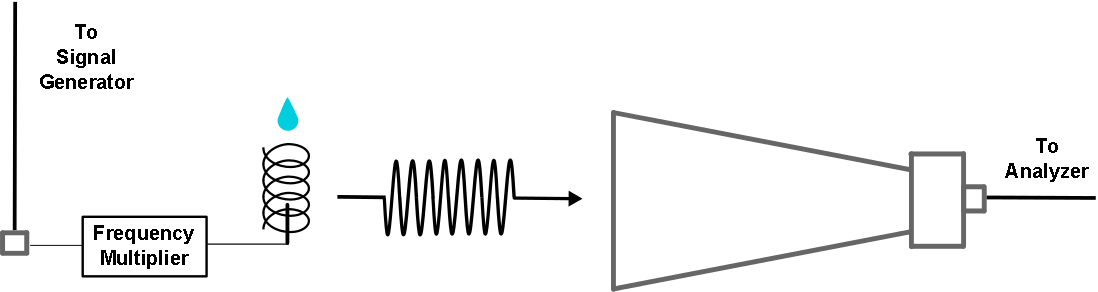

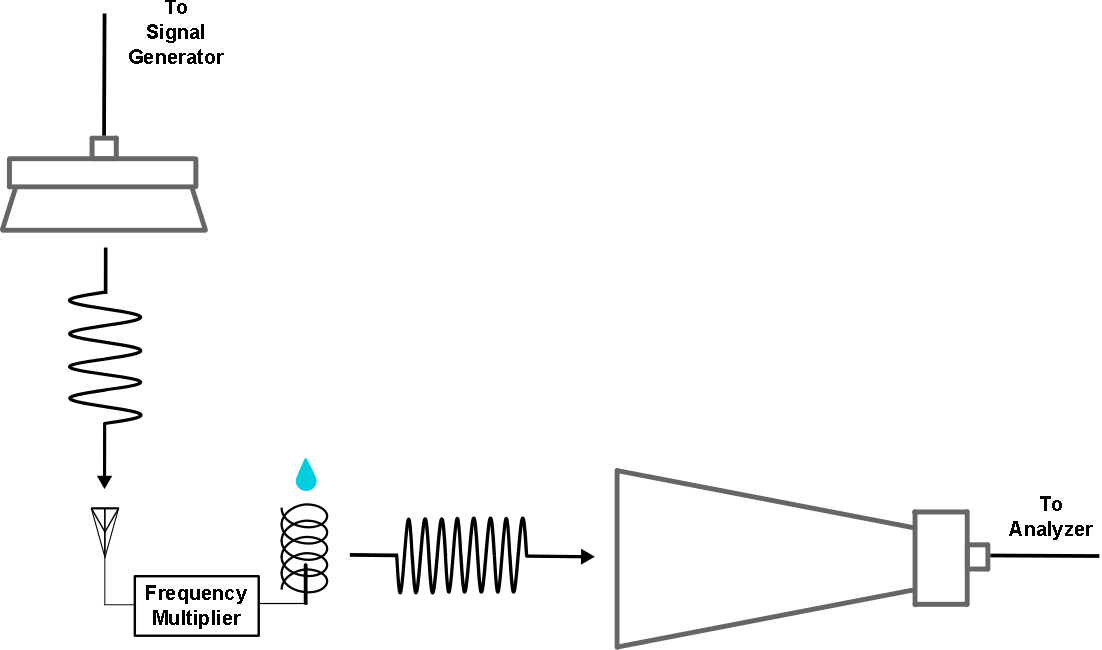


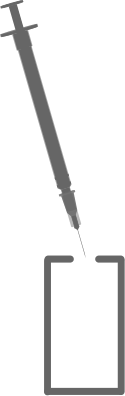


**b**

**a**


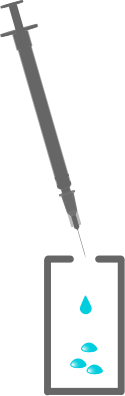

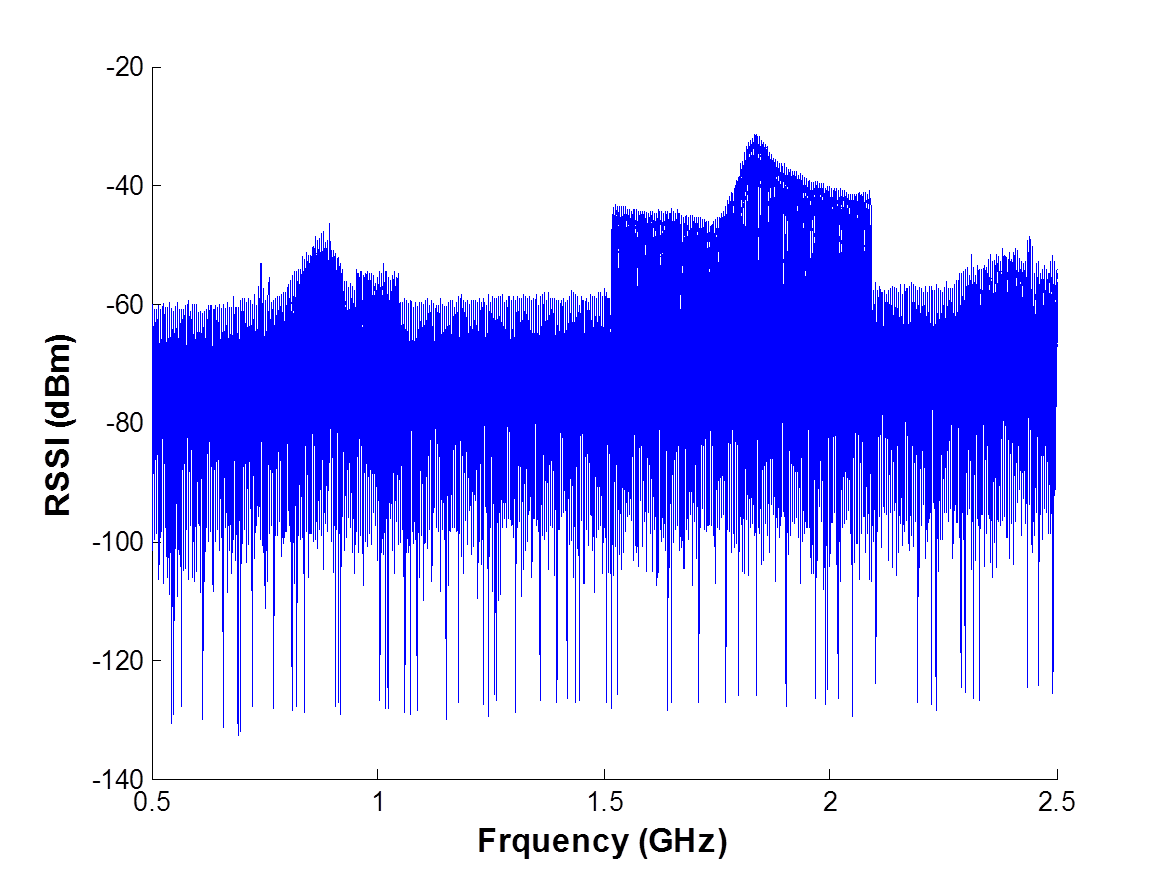

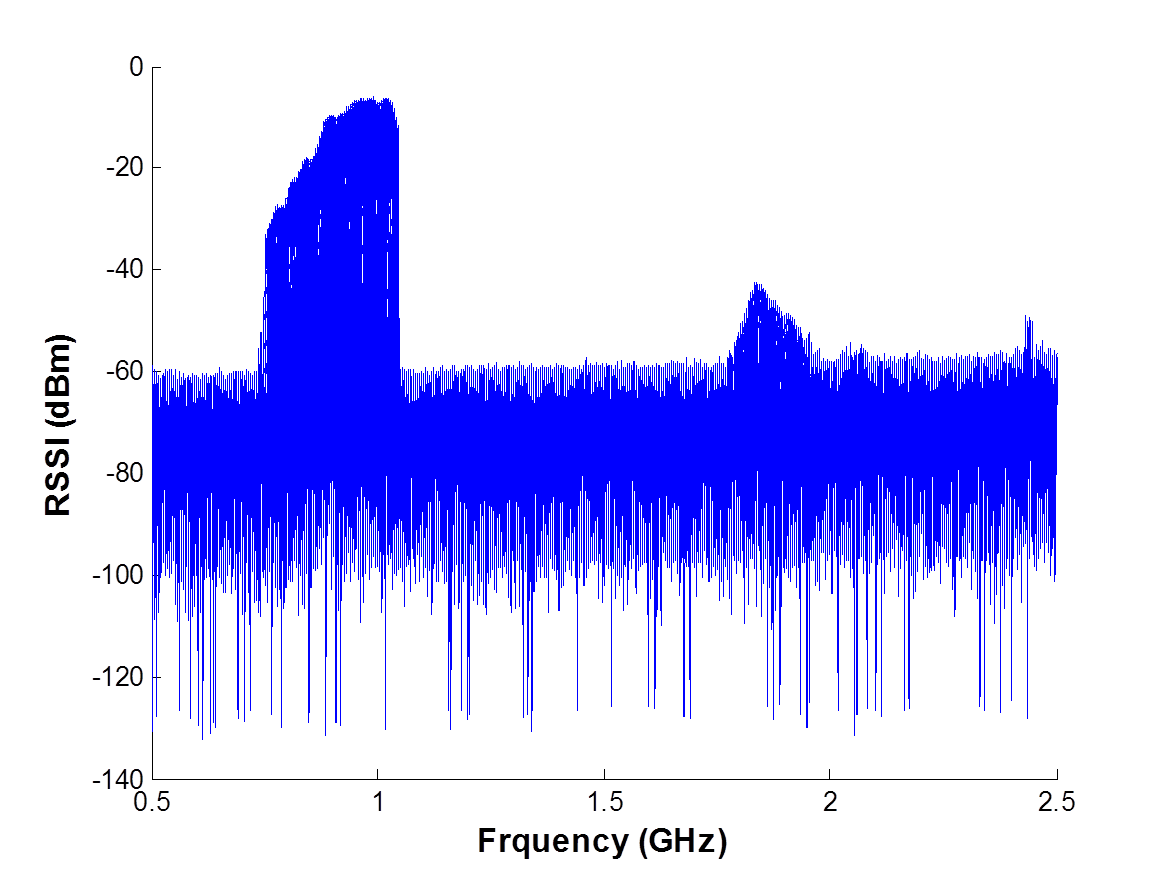


**d**

**c**


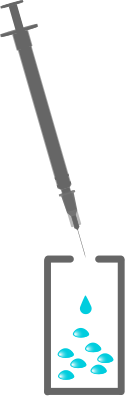

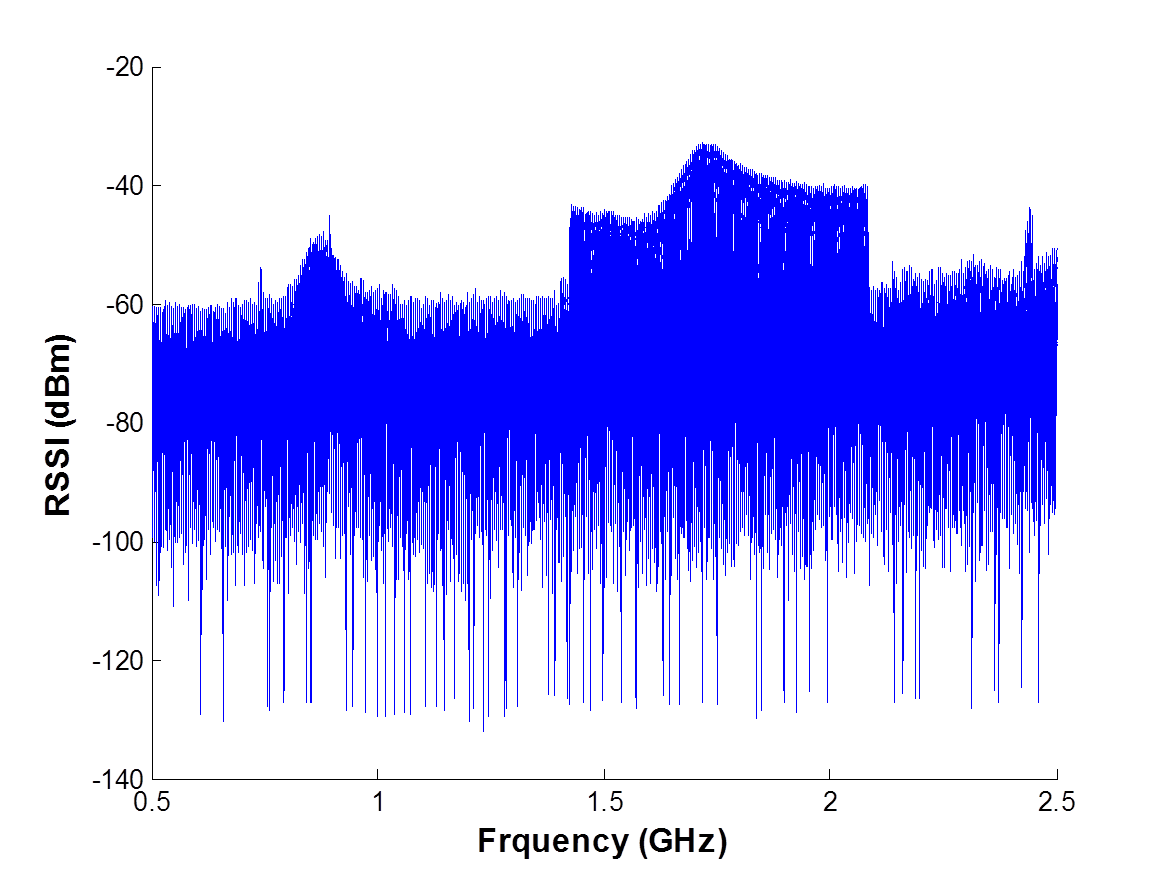

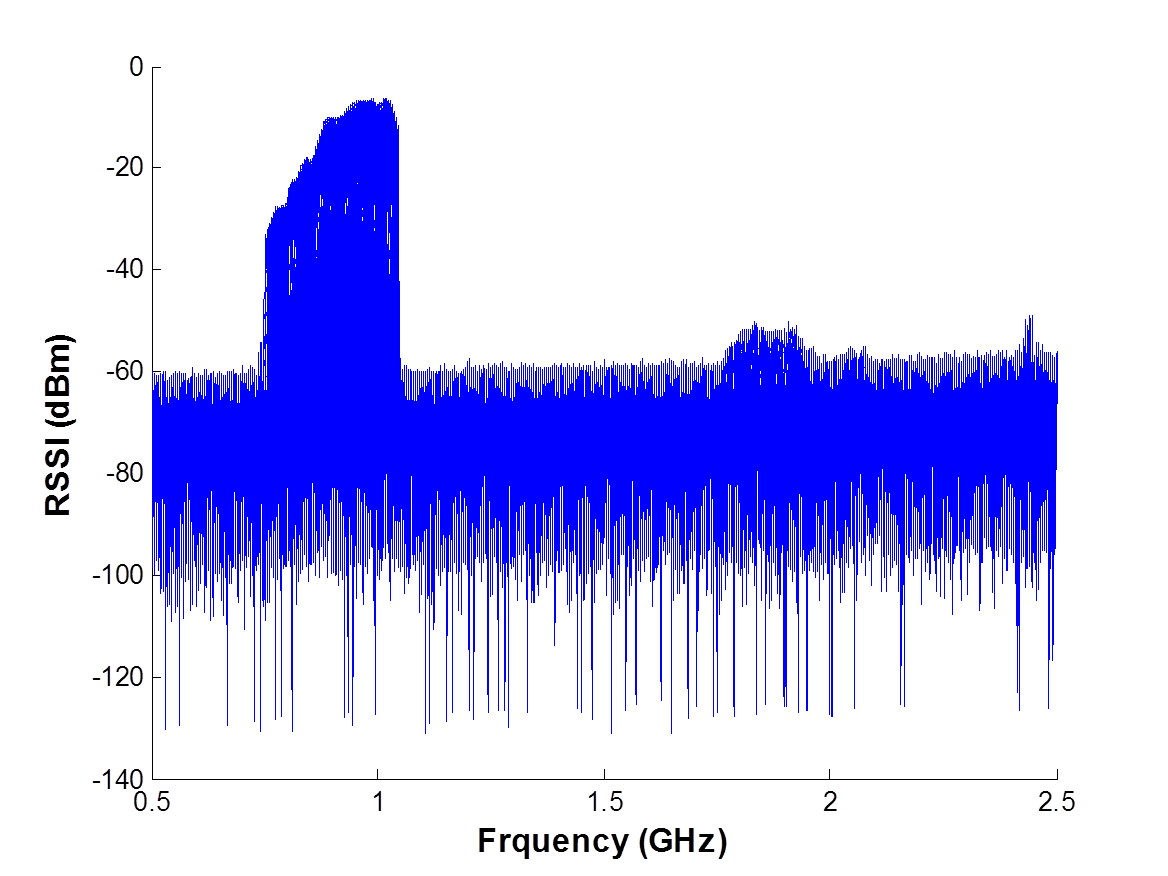


**f**

**e**


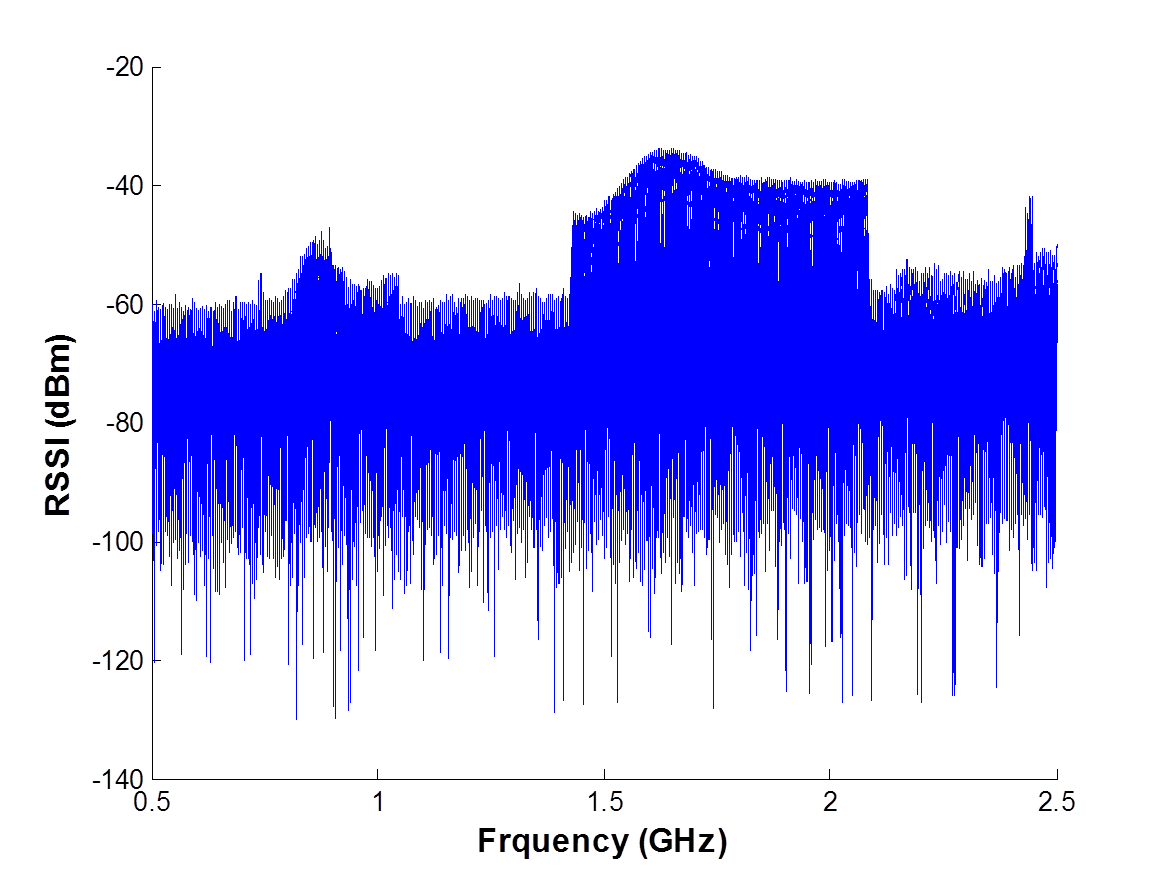

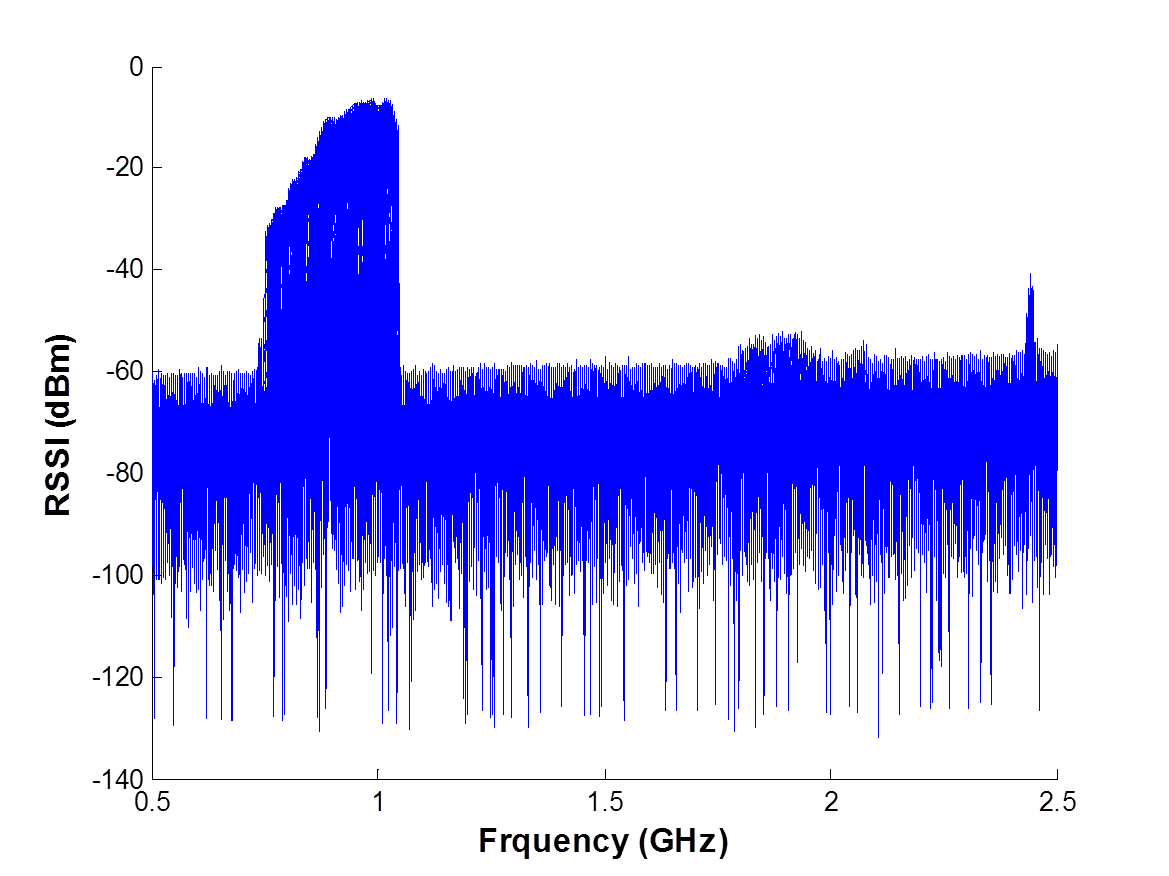


**Figure S5.** **Signal characterization with transponder liquid-sensitive antenna.** **(a)** Test of antenna sensor with frequency multiplier (with 25 cm transponder-to-sniffer distance) under frequency sweep from 700 MHz – 1.05 GHz; the harmonic peak is around 1.85 GHz with an empty reservoir. **(b)** Adding the interrogator and fundamental antenna at the transponder sensor for comparison (with 25 cm transponder-to-interrogator distance). The response under frequency sweep shows a peak around 1.85 GHz, which matches the peak in (a). **(c)** After applying 40 μL liquid to the reservoir, the peak of the antenna sensor response shifts to 1.72 GHz. **(d)** The complete passive wireless sensing system now shows a relatively weak and flat response due to the narrow band effect from the interrogator and fundamental antenna as shown in Fig. S2b. **(e)** After the liquid level adding up to 80 μL in the reservoir, the peak of the antenna sensor response further shifts to 1.62 GHz. **(f)** The complete wireless sensing system response does not change significantly comparing to (d), this can be explained by comparing (c) and (e), which have similar responses from 1.8 to 1.9 GHz as the peak already shifted toward much lower frequencies.


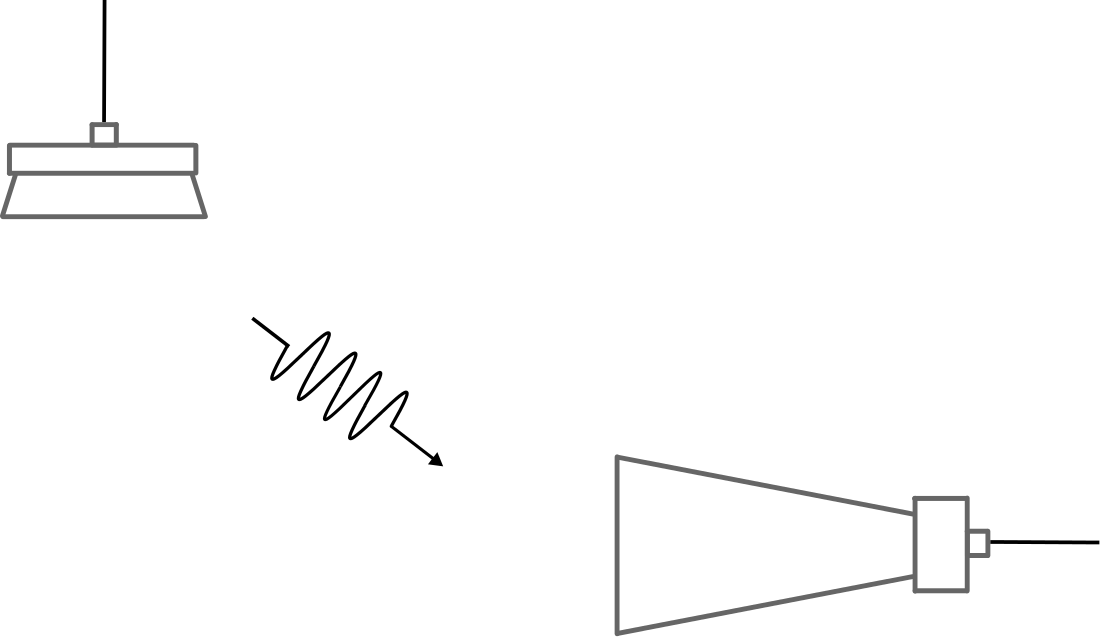

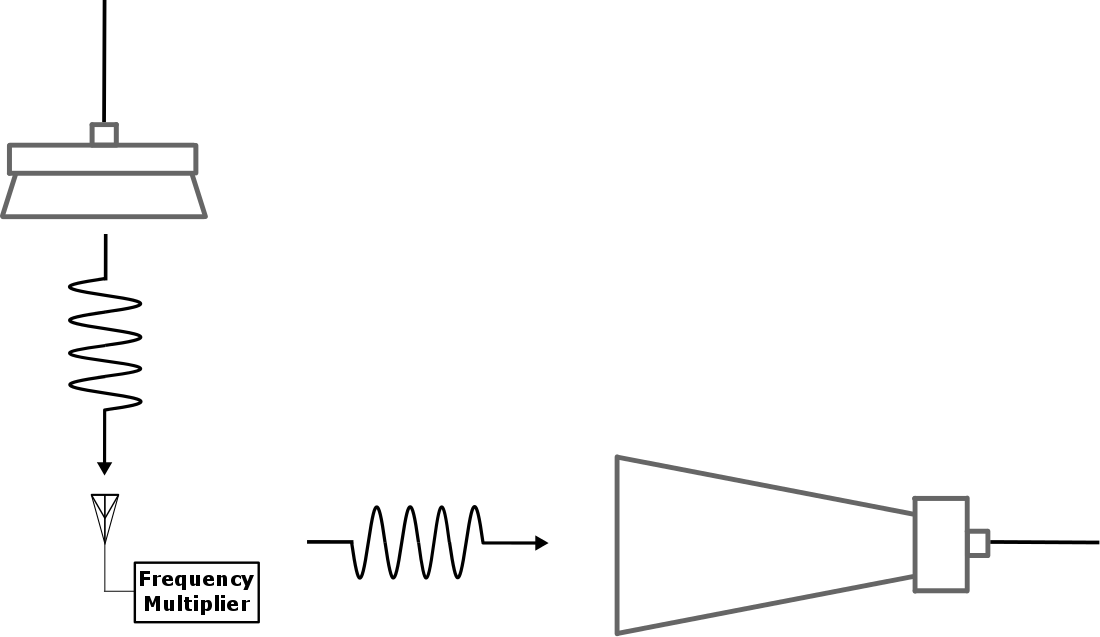

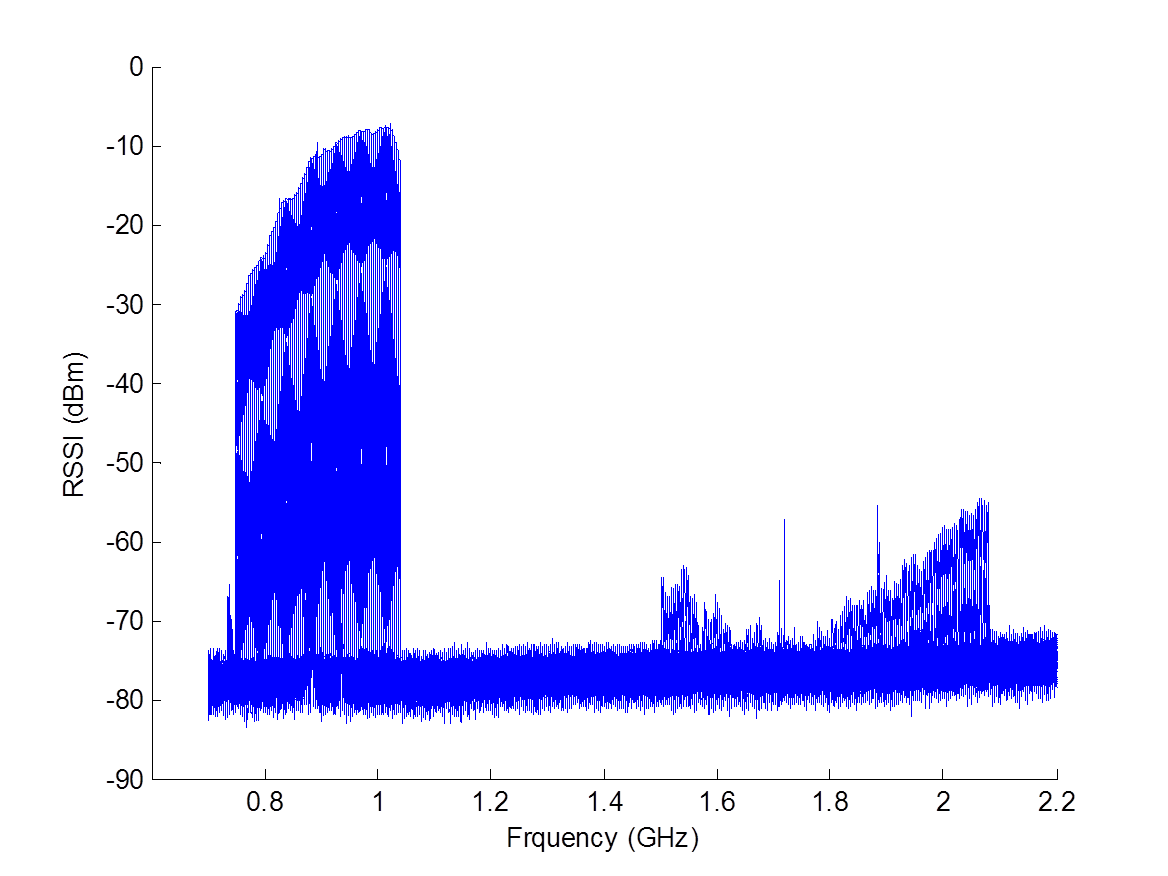

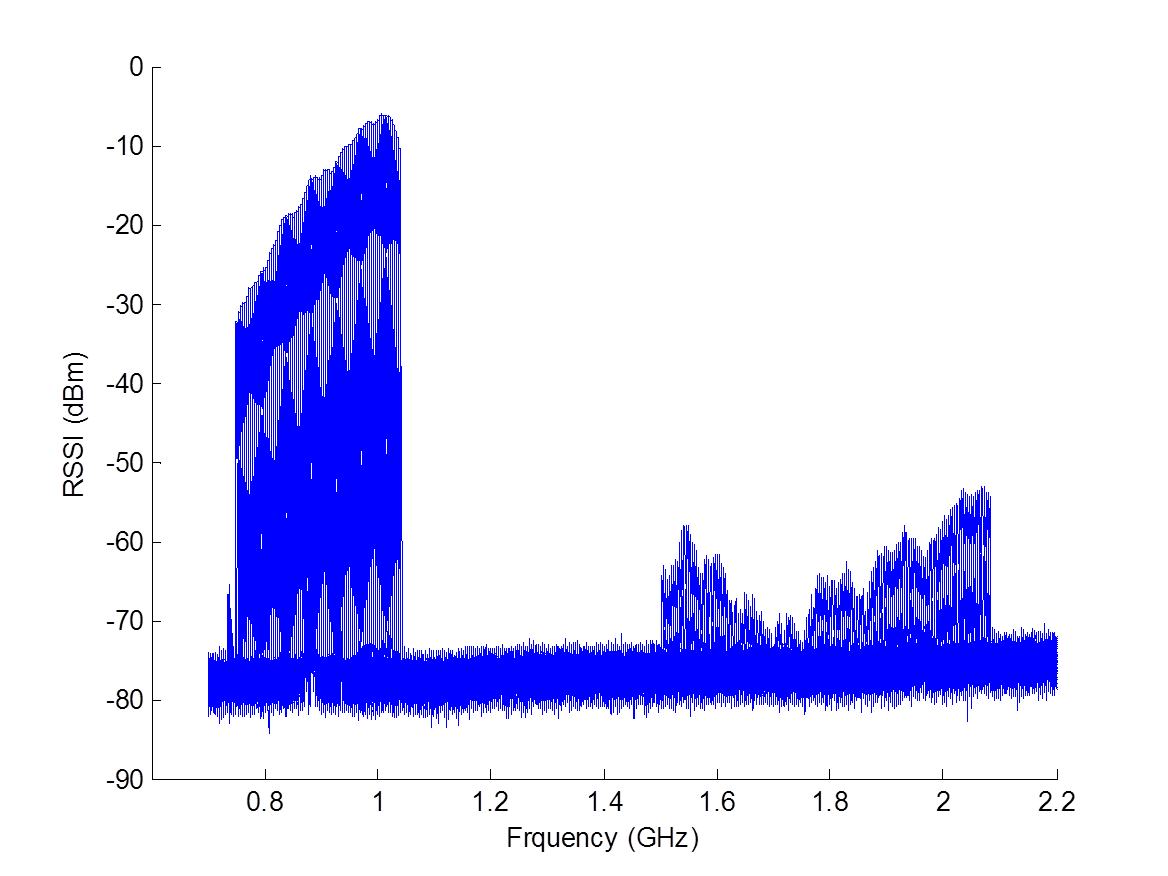


**b**

**a**

**d**

**c**


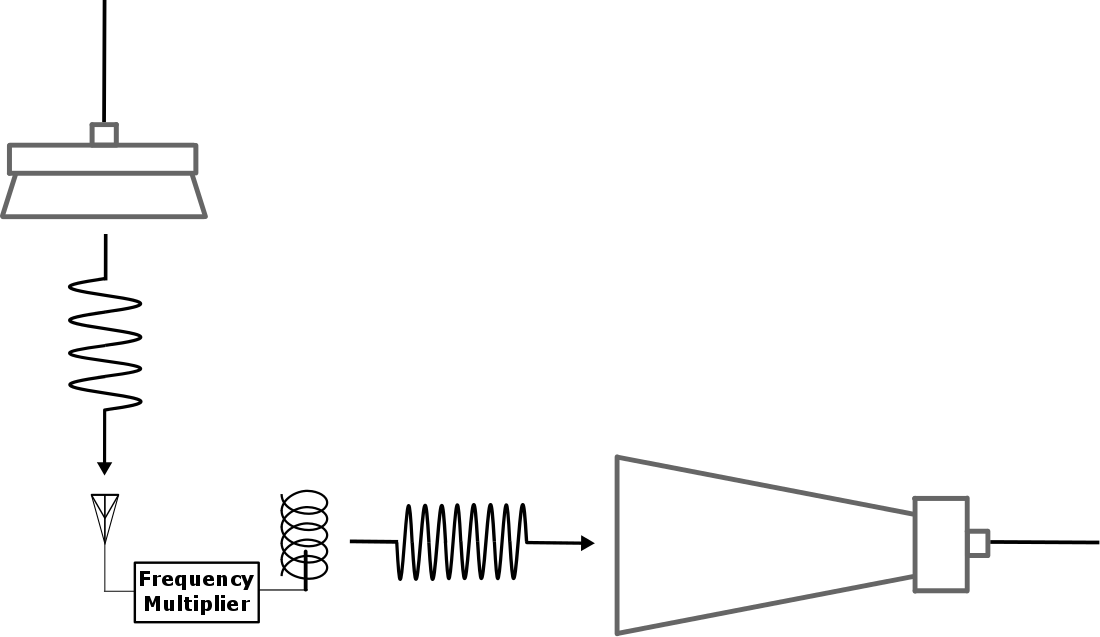

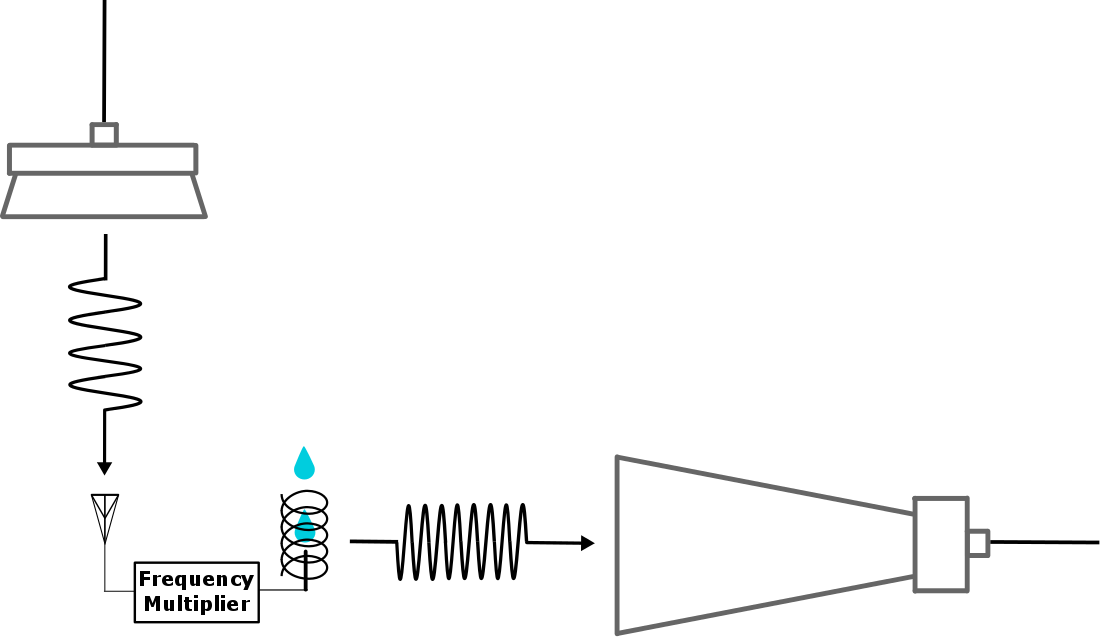

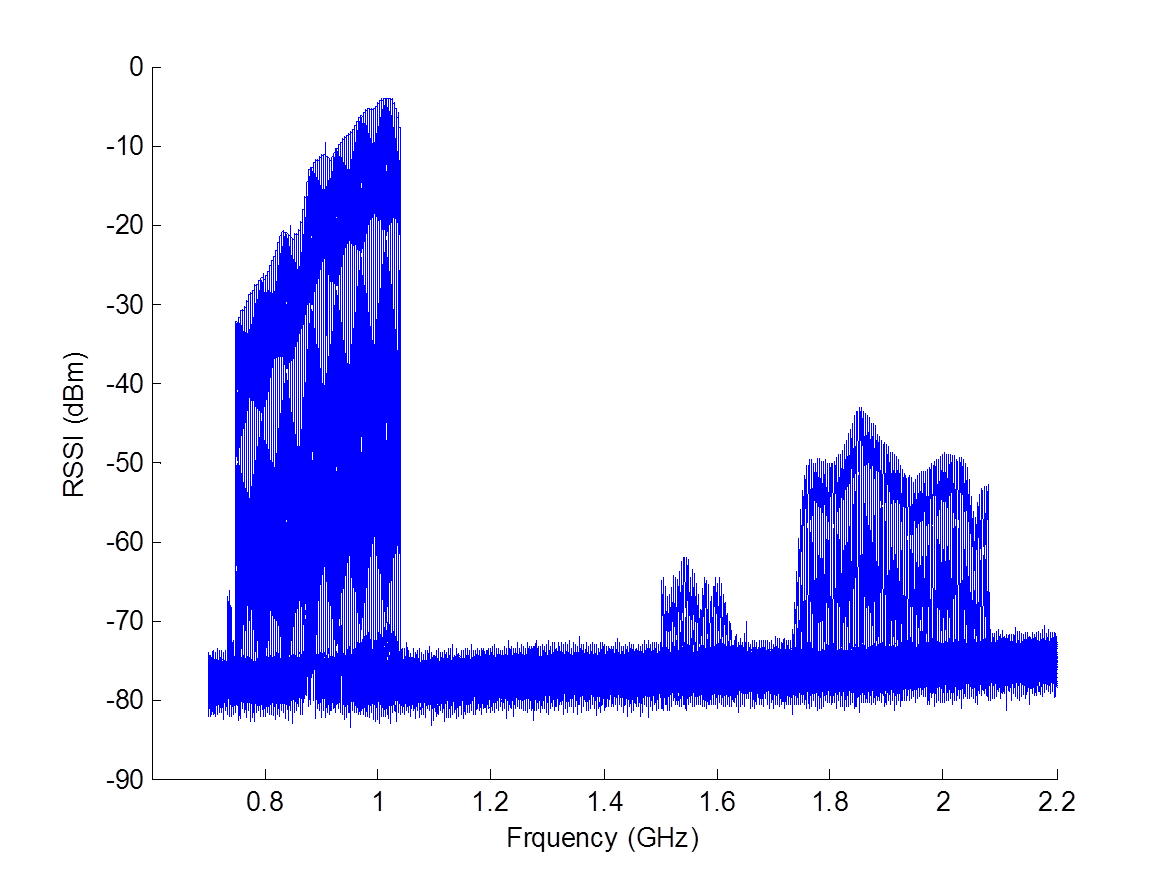

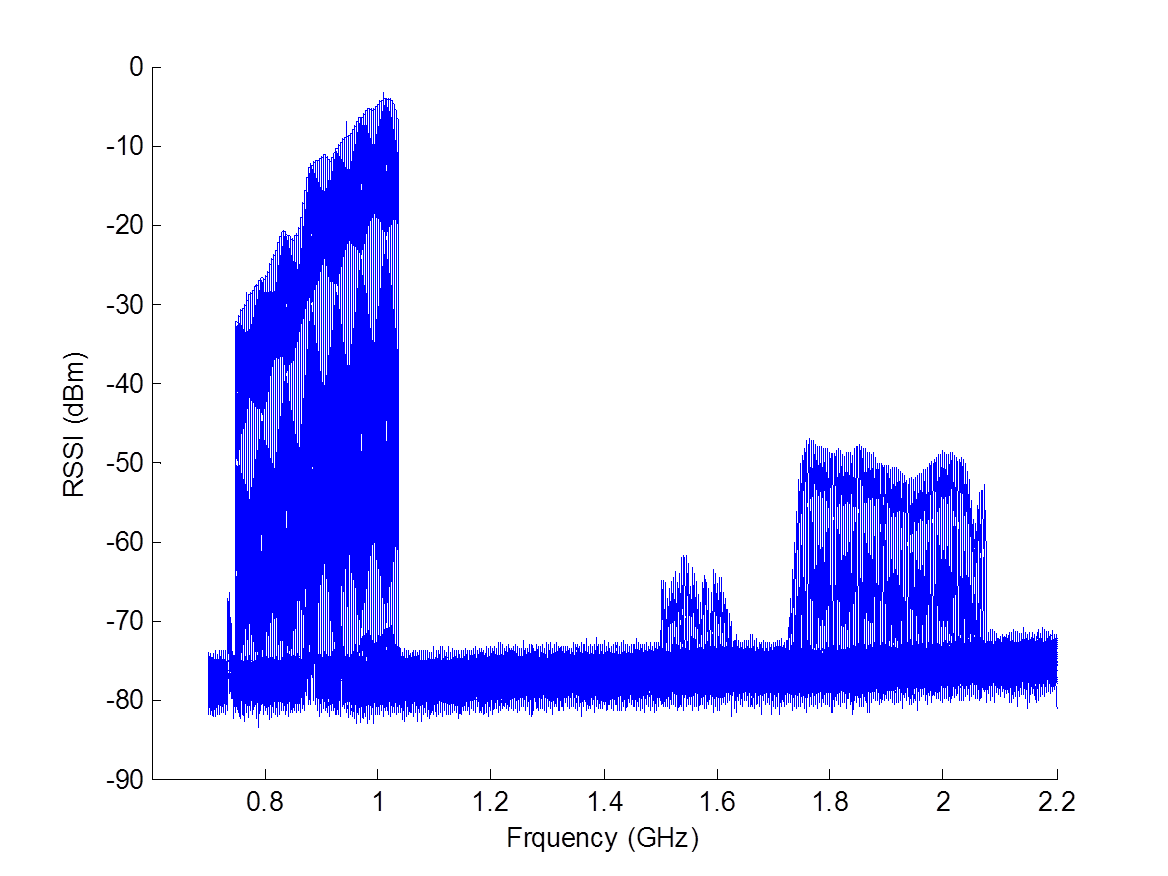


**Figure S6.** **Signal characterization for harmonic generation.** **(a)** Test of direct coupling from interrogator to wideband horn antenna sniffer (same relative position as in Fig.S5b) under frequency sweep from 750 MHz – 1.03 GHz with 0.5 MHz hopping channel width; the measured harmonic RSSI (with 100kHz resolution bandwidth) originated from the interrogator is below -70 dBm over the band of interest. **(b)** Adding the transponder without the antenna sensor, the harmonic RSSI is slightly affected by the backscatter. **(c)** Further adding the antenna sensor significantly increases the RSSI with a frequency hopped RSSI pattern peaked around 1.85 GHz. **(d)** Applying 30 μL to the sensor shifts the RSSI peak to 1.75 GHz.


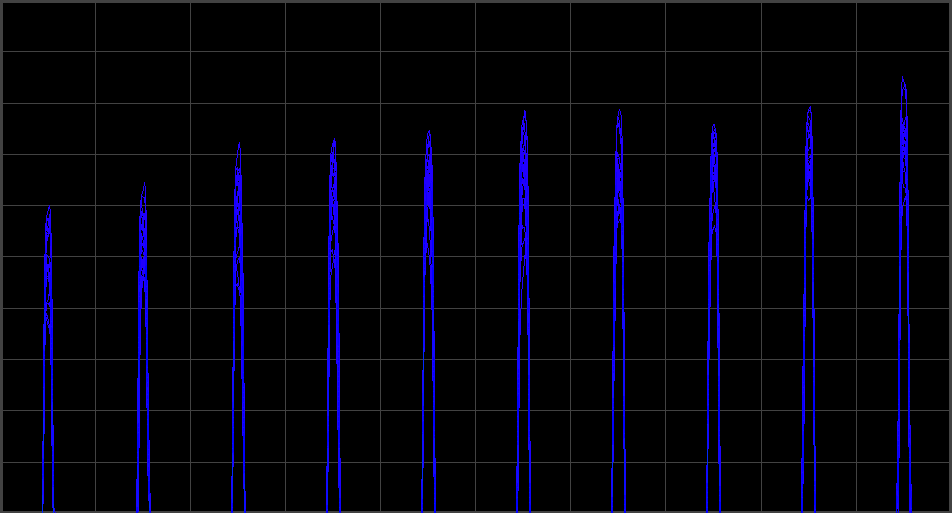

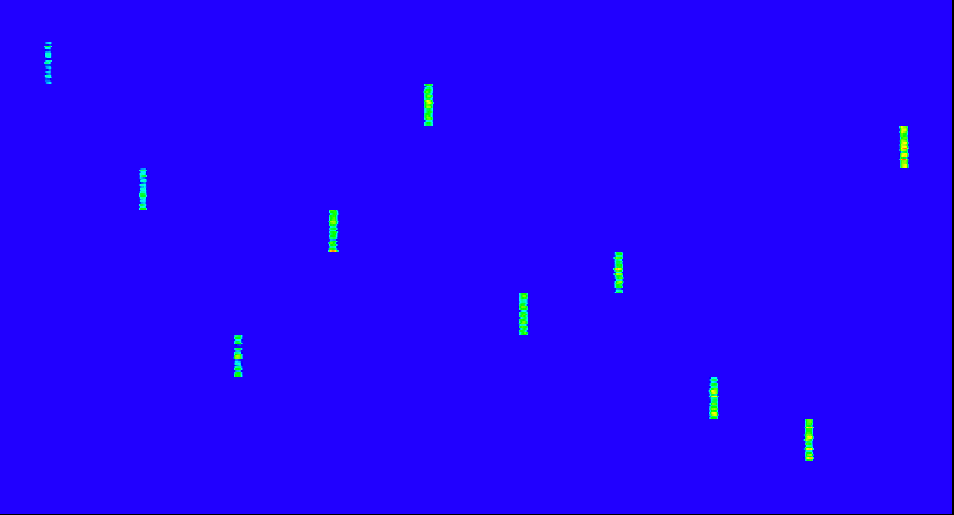

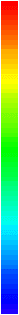


**b**

**a**

**d**

**c**


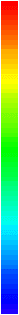

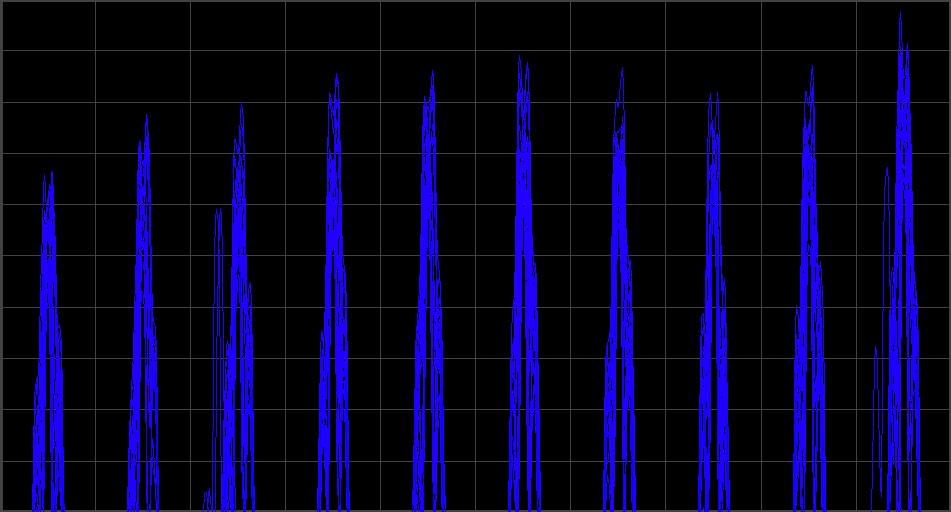

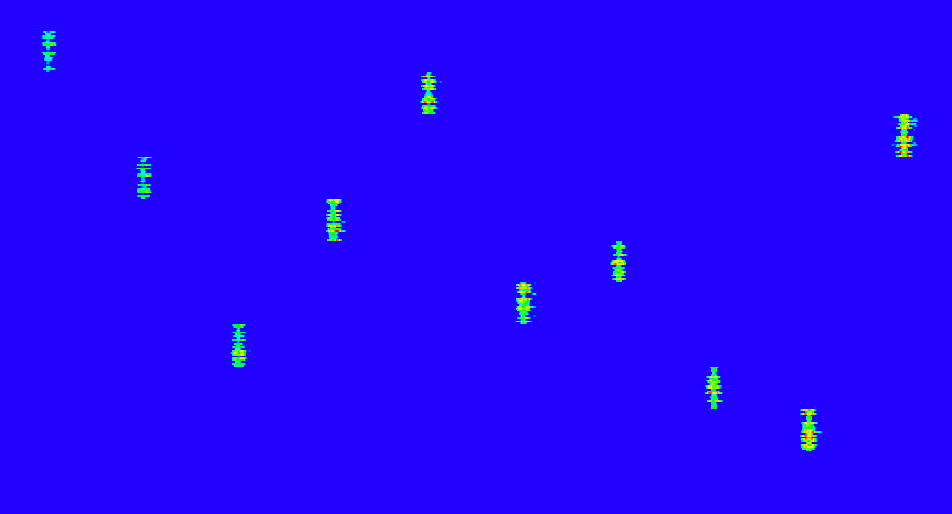


**f**

**e**

**h**

**g**


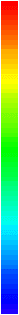

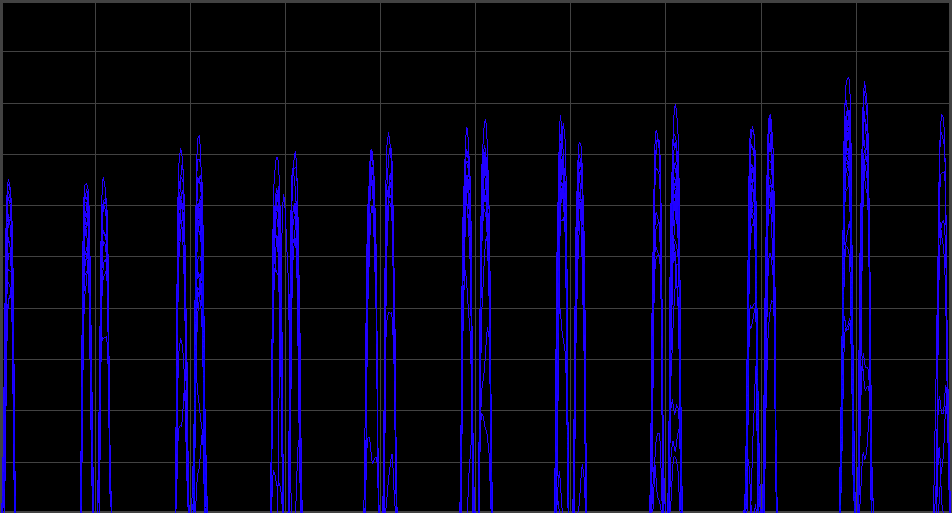

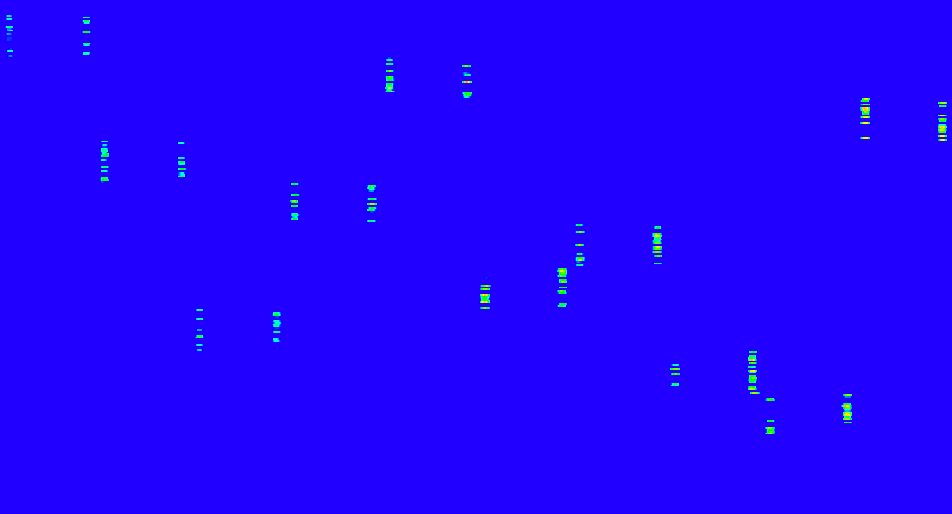


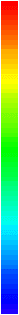

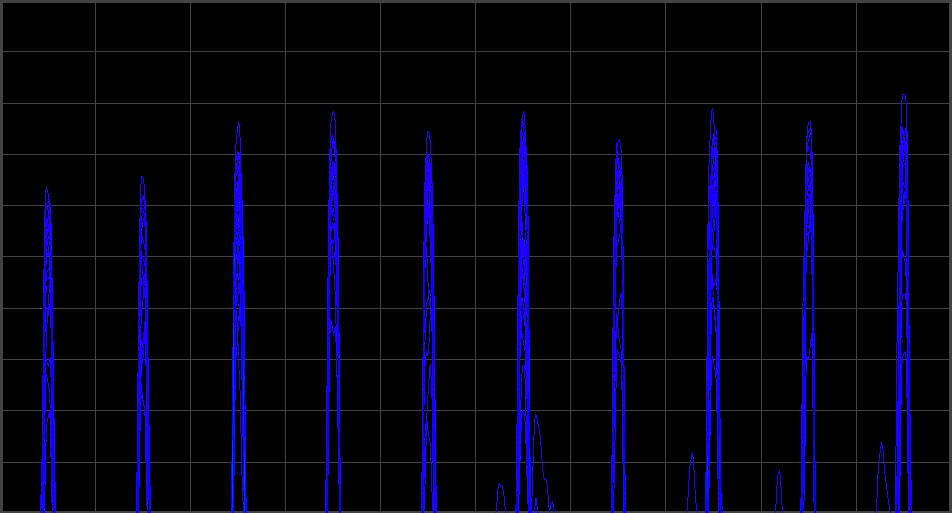

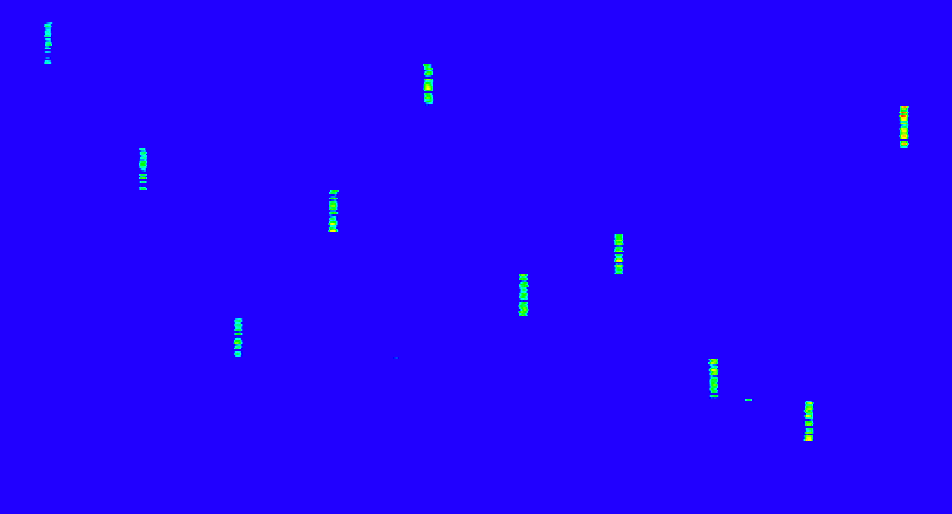


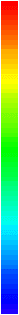

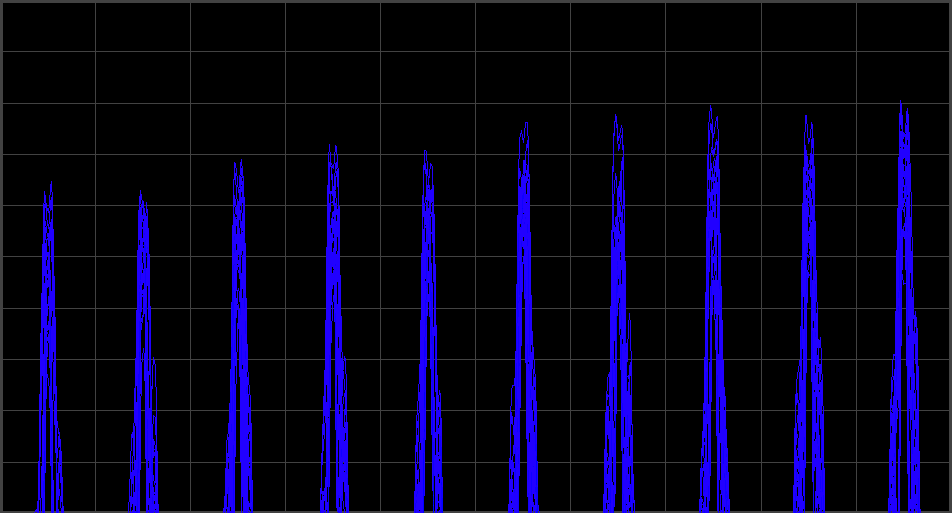

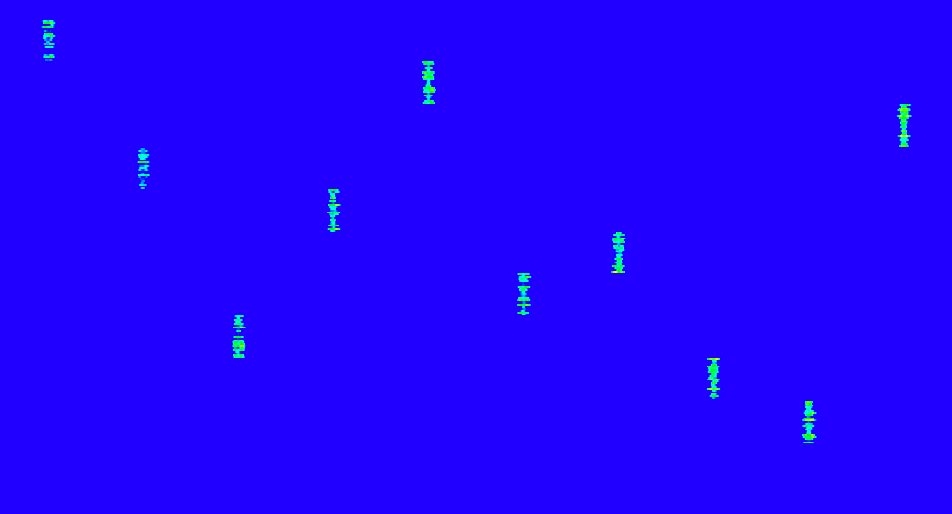


**j**

**i**

**l**

**k**


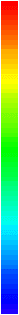

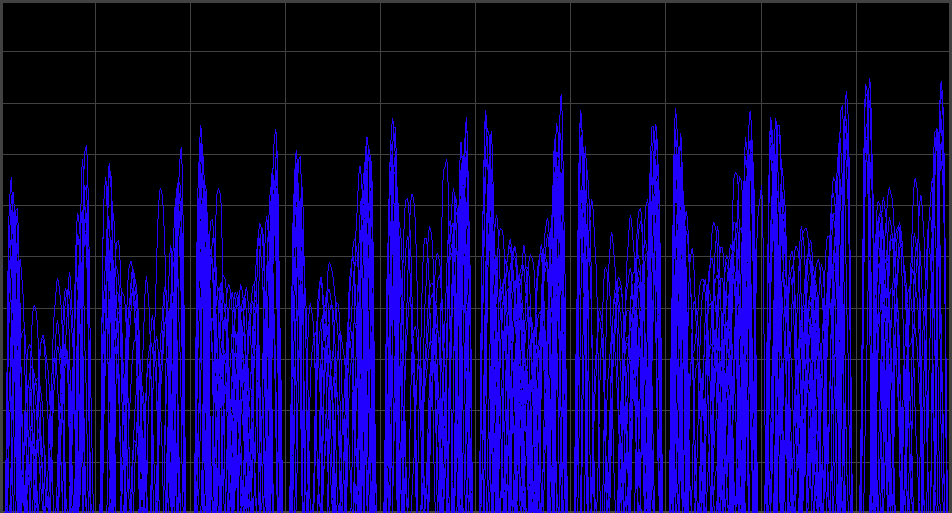

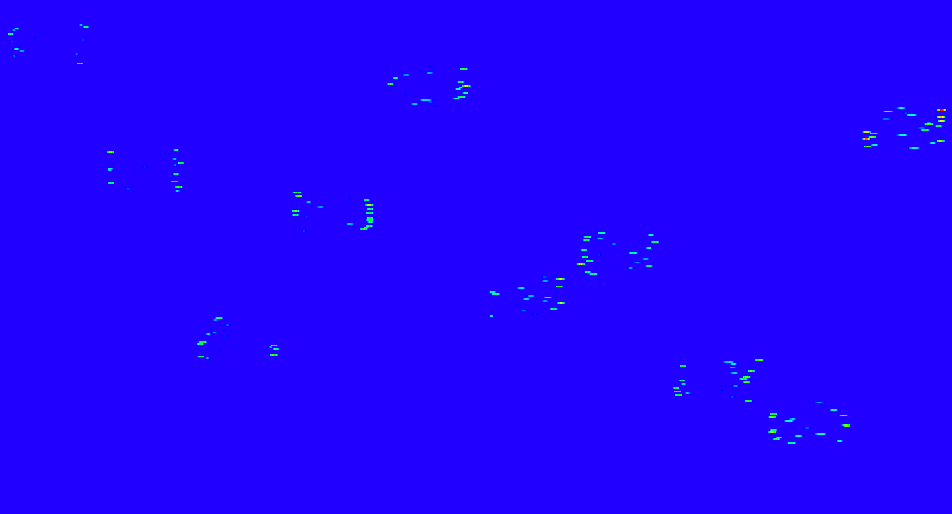


**Figure S7.** **Compatibility verification of FHSS harmonic transponder sensor with different digital and analog modulations on the interrogating signal source. The hopping range is set to 920 MHz to 925 MHz (1.84 GHz to 1.85 GHz harmonic band) among 10 channels; the spectrum analyzer resolution bandwidth is set to 100 kHz to better view signal modulation details. (a)** Spectrum density and **(b)** spectrogram of the received harmonic signal with single tone sinusoid. **(c)** Spectrum density and **(d)** spectrogram of the received harmonic signal with ASK signal (90% modulation depth with 100 kHz data rate). **(e)** Spectrum density and **(f)** spectrogram of the received harmonic signal with frequency shift key (FSK) signal (90% modulation depth with 100 kHz data rate). **(g)** Spectrum density and **(h)** spectrogram of the received harmonic signal with binary phase shift key (BPSK) signal (10 kHz data rate). **(i)** Spectrum density and **(j)** spectrogram of the received harmonic signal with amplitude modulation (AM) signal (90% modulation depth with 100 kHz data rate). **(k)** Spectrum density and **(l)** spectrogram of the received harmonic signal with frequency modulation (FM) signal (90% modulation depth with 100 kHz data rate).

**Video S1.** **FHSS Pattern analysis based sensor readout.** The process of frequency hopping and construction of FHSS pattern. Right liquid-volume sensing decisions can be made within only a few hops, showing strong robustness and real-time sensing capability.

**References:**

1. Ziolkowski, R. W. Efficient electrically small antenna facilitated by a near-field resonant parasitic. *IEEE Antennas Wirel. Propag. Lett.* **7,** 581–584 (2008).

2. Liao, Y., Hubing, T. H. & Su, D. Equivalent Circuit for Dipole Antennas in a Lossy Medium. *IEEE Trans. Antennas Propag.* **60,** 3950–3953 (2012).

3. Su C., K. H. H. T. A simplified model for Normal mode helical antennas. *Appl. Comput. Electromagn. Soc. J.* **25,** 32–40 (2010).

4. Advanced Design System (ADS) | Keysight (formerly Agilent Test and Measurement). at <http://www.keysight.com/en/pc-1297113/advanced-design-system-ads?cc=US&lc=eng>
